# Nucleosome linker DNA methylation by DNMT3A/DNMT3B3 is controlled by nucleosome binding and multimerization of DNMT3 complexes on DNA

**DOI:** 10.1016/j.jbc.2026.111154

**Published:** 2026-01-10

**Authors:** Nicole Gutekunst, Alexander Bröhm, Pavel Bashtrykov, Albert Jeltsch

**Affiliations:** Institute of Biochemistry, University of Stuttgart, Stuttgart, Germany

**Keywords:** DNMT3A, DNA methyltransferase, nucleosome, enzyme catalysis, enzyme mechanism, linker DNA, DNA methylation

## Abstract

Structural and biochemical studies showed that DNMT3A/DNMT3B3 (3A/3B3) heterotetramers directly interact with the nucleosomal acidic patch *via* the DNMT3B3 subunit. Here, we investigated linker DNA methylation by 3A/3B3 using dinucleosome substrates as most suitable mimic of linker chromatin methylation in cells. Dinucleosomes with different linker lengths and sequence were used and DNA methylation was investigated quantitatively by bisulfite sequencing. The effects of nucleosomal recruitment were investigated using 3A/3B3 C-terminal domain complexes and complexes containing the R740E/R743E double mutation in DNMT3B3 which affects the two most important residues in the DNMT3B3-acidic patch contact. Using competitive methylation assays of nucleosomal and free DNA, we demonstrate that the contact to the acid patch improves 3A/3B3 recruitment to nucleosomes and methylation of linker DNA. Characteristic methylation levels of CpG sites next to the nucleosomes suggest that 3A/3B3 complexes are anchored on both sides of the linker DNA to nucleosomes. However, detailed analysis of linker DNA methylation levels revealed nucleosome dependent methylation patterns even at CpG sites that are not in direct proximity to the nucleosomes suggesting that DNMT3A complexes multimerize on the linker DNA. This multimerization spatially organizes the complexes, aligning active sites of DNMT3A complexes with CpG sites, which then leads to the observed methylation patterns. Moreover, product DNA molecules with high methylation levels were strongly overrepresented also indicating that DNMT3A fiber formation leads to cooperative linker DNA methylation. Our data suggest that multimerization of DNMT3A on linker DNA could shape the DNA methylation landscape in cells with potential implications on nucleosome positioning particularly in heterochromatic regions.

DNA methylation is an evolutionarily conserved epigenetic gene regulatory mechanism that critically influences chromatin structure and function (1, 2). In mammals, DNA methylation has essential roles in normal development and diseases (3, 4, 5). It predominantly occurs at the C-5 position of cytosines in CpG sequences which are methylated at about 75 to 80% genome-wide in human tissues (6, 7). DNA methylation is mainly established by the *de novo* DNA methyltransferases DNMT3A and DNMT3B during gametogenesis and embryogenesis and maintained after DNA replication by DNMT1 (8). However, the DNMT3 enzymes are also required for the long-term maintenance of DNA methylation in somatic cells. DNMT3A and DNMT3B are large multidomain proteins that are expressed in different splicing isoforms (9, 10), but their C-terminal domains are catalytically active in isolated form (11). DNMT3L is a catalytically inactive paralog of the DNMT3 enzymes mainly expressed in germ cells that stimulates the catalytic activities DNMT3A and DNMT3B (9). It lacks parts of the N-terminal region and contains mutations in the C-terminal part that render it catalytically inactive. DNMT3B3 is a catalytically inactive splicing isoform of DNMT3B that is expressed in differentiated cells and tumors and stimulates DNMT3A in a manner reminiscent of DNMT3L (12). Structural and biochemical work revealed that all DNMTs flip their target base out of the DNA helix to catalyse its methylation (9, 13). In recent years, it has been discovered that DNMT3A and DNMT3B methylate DNA with strong and characteristic preferences for at least 3 bp sequences flanking the CpG site and these preferences have strong effects on cellular methylation patterns (14, 15, 16, 17, 18).

Structural studies have shown that the catalytic domains of DNMT3A (DNMT3AC) and DNMT3B contain two protein/protein interfaces called RD and FF interface (Fig. 1*A*) (15, 19). Therefore, these proteins can multimerize forming large protein fibers (Fig. 1*B*) (20, 21, 22, 23, 24, 25). Due to deletions in the RD domain, the corresponding C-terminal domains of DNMT3L (DNMT3LC) and DNMT3B3 (DNMT3B3C) only contain a functional FF-interface (Fig. 1*A*) which limits the multimerization of DNMT3A/DNMT3L and DNMT3A/3B3 complexes (Fig. 1*B*) to linear heterotetramers with two DNMT3A subunits in the center and one DNMT3L or DNMT3B3 subunit at each edge (19, 26, 27). The two central DNMT3A subunits of these complexes harbor two active sites that can simultaneously interact with two CpG sites on one DNA molecule ideally separated by 12 bp and co-methylate the top strand of the first and bottom strand of the second CpG site (16, 26). In addition, DNMT3 complexes have been shown in biochemical and structural studies to bind to and methylate DNA cooperatively (28, 29). During this process, DNMT3 complexes assemble next to each other on the DNA forming a protein-DNA fiber as shown by atomic force microscopy imaging (16, 21, 22, 29), and adjacent complexes are able to methylate CpG sites in 6 bp distance (16) (Fig. 1*C*). The interface between two DNMT3 complexes bound next to each other on the DNA in one possible conformation has been mapped and was found to be distinct from the FF and RD interfaces (29).

**Figure 1:**
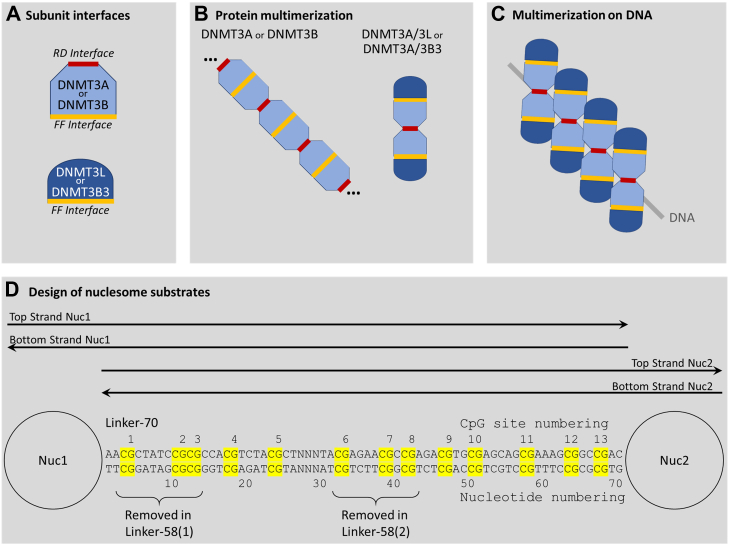
**Schematic drawing of the multimerization modes of DNMT3 complexes and of the dinucleosome substrates used in this study.***A*, protein/protein interaction interfaces present on DNMT3A, DNMT3B, DNMT3L, and DNMT3B3 subunits. The RD interface also provides the DNA binding site. *B*, protein multimerization of different DNMT3 complexes. DNMT3A and DNMT3B can form large homomultimers; homotetramers with one central RD interface are the smallest catalytically active species. DNMT3A/3L and DNMT3A/3B3 form defined heterotetramers. *C*, multimerization of DNMT3 complexes binding next to each other on DNA. *D*, dinucleosomes used as methylation substrates in this study. The CpG sites in the linker DNA region are highlighted and annotated. The regions used for bisulfite sequencing of the *top* and *bottom* DNA strand are indicated. The parts of the 70 bp linker removed in the 58(1) and 58(2) linkers are indicated. The NNN part between CpG 5 and CpG 6 denotes an internal bar code, used to discriminate the NGS data from pooled substrates. NNN = TGT in Linker-70, TCA in Linker-58(1), CTA in Linker-58(2). NGS, next generation sequencing.

The cryo-EM structure of a DNMT3A2/DNMT3B3 heterotetramer bound to a mononucleosome revealed that one of the distal DNMT3B3 subunits directly binds the H2A/H2B acidic patch on the histone octamer *via* R740 and R743, thereby anchoring the DNMT3A2/3B3 tetramer on the nucleosome core particle (27). Methylation of nucleosomal DNA by different DNMT3A complexes, including DNMT3A2 and DNMT3AC homotetramers, and DNMT3AC/3B3C heterotetramers, showed strong protection of the nucleosomal bound DNA from methylation (30, 31, 32, 33). A detailed analysis of the methylation activities of DNMT3A complexes on linker DNA revealed that the methylation of two specific CpG sites in the linker DNA region next to the nucleosome core (sites #12 and 13 in the current work) agreed very well with the structural details of the cryo-EM structure suggesting that the nucleosome docked conformation of DNMT3A is relevant under enzymatic conditions (33). Comparison of the data obtained with different DNMT3A complexes suggested that DNMT3A2 and DNMT3AC homotetramers form a similar interaction with the nucleosome as DNMT3B3 (33). However, strong methylation of the linker DNA was also observed at CpG sites far away from the nucleosome (33) which is indicative of a second mechanism of DNMT3A recruitment to the linker DNA that is independent of the direct nucleosome contact. Whether this is due to a flexible movement of the anchored DNMT3A complex on the linker DNA, multimerization of DNMT3A complexes on the DNA starting at the anchored complex, or binding of freely diffusing DNMT3A complexes independent of the one anchored to the nucleosome remained unclear. Moreover, the specific kinetic effects of the DNMT3A/3B3 recruitment to nucleosomes on linker DNA methylation had not been determined so far.

It was the aim of this study to resolve these mechanistic questions by investigating the catalytic activities of DNMT3A/3B3 complexes on dinucleosome substrates. To directly investigate the effects of the DNMT3B3-acidic patch contact, complexes containing the R740E/R743E double mutation in DNMT3B3 were used, which affect the two most important residues in this contact. Our data show that the DNMT3B3 contact to the acid patch improves DNMT3A/3B3 recruitment to nucleosomes and methylation of linker DNA. Moreover, our data indicate that DNMT3A/3B3 complexes are anchored on both sides of the linker DNA. These contacts trigger the multimerization of DNMT3A complexes on the linker DNA leading to distinct nucleosome dependent methylation patterns along the entire linker DNA. Based on our findings, multimerization of DNMT3A on linker DNA could potentially contribute to DNA methylation and nucleosome positioning in cells especially in heterochromatic regions.

## Results

Chromatin bound DNA is an important physiological substrate of DNA methyltransferases. Previous work showed that DNMT3A and other DNMTs are inactive on nucleosomal bound DNA, but they methylate linker DNA regions (30, 31, 32, 33). Indeed, cellular DNA methylation analyses revealed higher DNA methylation levels in linker DNA regions than in nucleosome core regions (34). To study the detailed mechanism of nucleosomal linker DNA methylation in a physiologically relevant setting, we investigated the methylation of dinucleosomal DNA substrates, which contain linker DNA capped by nucleosomes on both ends thereby mimicking chromatin substrates in cells. To obtain detailed information about the linker DNA methylation patterns, DNA methylation was analyzed quantitatively by bisulfite sequencing in both DNA strands with CpG site resolution. The consequences of nucleosomal recruitment of DNMT3 complexes on the methylation of individual CpG sites were studied using three dinucleosome substrates with different linker lengths and sequence. The kinetic effects of the recruitment of DNMT3A/3B3 to the nucleosome acidic patch were investigated using DNMT3AC/3B3C heterotetrameric complexes and complexes containing the R740E/R743E double mutation in DNMT3B3C (now called RE mutant), which contains two charge reversal mutations at critical amino acid residues that were shown to disrupt the DNMT3B3-acidic patch contact (27).

### Generation of dinucleosomes and purification of DNMT3A tetramer complexes

To generate recombinant dinucleosomes, DNA templates were prepared containing two Widom-601 nucleosome binding sites (35) that were separated by a linker DNA of 70 bp containing 13 CpGs to provide space for the methylation (Linker-70) (Fig. 1*D*). In addition, dinucleosome DNA templates were prepared with two types of 58 bp long linkers, a linker length that is characteristic for heterochromatic chromatin domains containing H3K9me3 and H4K20me3 or H3K27me3 (36). For this, 12 bps containing CpG site 1 to 3 were removed in Linker-58(1) and 12 bps containing CpG 6 to 8 in Linker-58(2) (Fig. 1*D*). Dinucleosome reconstitution started with purified DNA and histone octamers, and it was validated by gel retardation assays (Fig. S1). For the generation and purification of DNMT3AC/3B3C and DNMT3AC/3B3 mutant heterotetramers, maltose-binding protein (MBP)-tagged DNMT3A containing a tobacco etch virus (TEV) cleavage site and His-tagged DNMT3B3C (or DNMT3B3C mutant) were co-expressed in *Escherichia coli* and the heterotetramers purified by two successive purification steps for the two affinity tags (37) (Fig. S2*A*). Purified proteins are shown in Figure S2*B*. Prior to the methylation reactions, the MBP-tag was removed by protease treatment (Fig. S2*C*).

### Methylation of dinucleosome substrates by DNMT3AC/3B3C

The three different dinucleosome substrates were methylated with DNMT3AC/3B3C, the DNA recovered, bisulfite converted, and methylation analyzed in both DNA strands by amplicon based next generation sequencing (NGS) (38). For this, the Nucleosome 1–Linker–Nucleosome 2 target sequence was split into two sequencing amplicons, one comprising the Nucleosome 1–Linker and the second the Linker–Nucleosome 2 region (Fig. 1*D*). Methylation reactions were conducted in five experimental replicates which were normalized to their average linker DNA methylation yielding relative methylation levels of the CpG sites. The DNA methylation levels obtained with the two different sequencing amplicons at corresponding linker CpG sites were very similar in all data sets with most sites showing deviations of <20% (Fig. S3*A*). Therefore, the linker DNA methylation data were averaged to obtain the final data sets with five data points based on independent experimental repeats for each CpG site. As control to assess the rate of incomplete conversion in the bisulfite treatment, reactions were prepared without enzyme and analyzed using the same workflow for all experiments. As expected, the false positive methylation levels were low in the range of 1 to 1.5% per CpG site (Fig. S3*B*). This residual level of incomplete conversion of unmethylated cytosine residues is expected for the bisulfite sequencing technology. We conclude that the overall workflow is functional and reliable.

**Figure 2:**
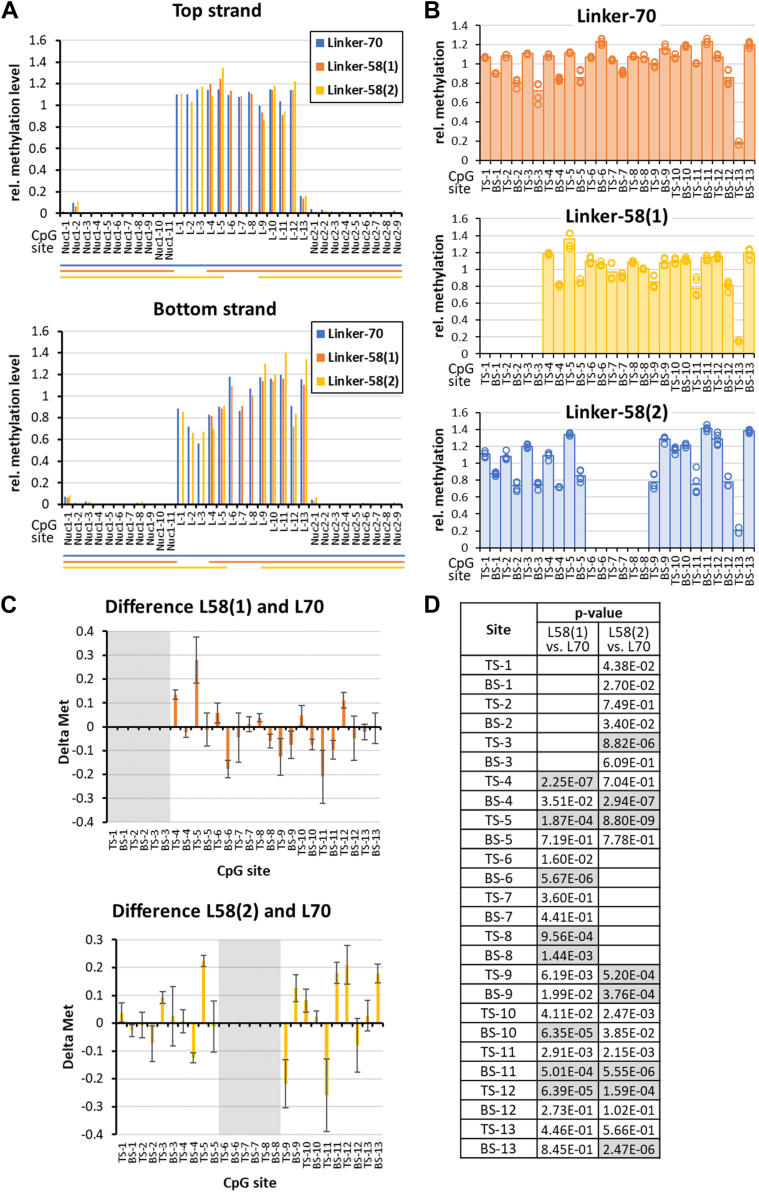
**Linker methylation patterns of all CpG sites in the three dinucleosomes by DNMT3AC/3B3C.***A*, example of *top* strand and *bottom* strand methylation data. Values were normalized to the average methylation of linker CpG sites. *B*, relative linker methylation levels observed in five independent experiments. In *panel A* and *B*, no methylation data were indicated for the missing CpG sites 1 to 3 in Linker-58(1), and 6 to 8 in Linker-58(2). *C*, difference in the relative methylation levels of equivalent CpG sites between Linker-70 and Linker-58(1) or 58(2). The regions missing in the Linker-58 nucleosomes are shaded in *gray*. Error bars represent the propagated standard deviations. *D*, *p* values for the significance of the methylation differences shown in *panel C* determined by two-sided *t* test with equal variance using the individual data points. Significant *p* values (*p* < 0.05/26, considering multiple testing) are shaded in *gray*.

The methylation data of all three dinucleosomes showed strong methylation in the linker regions and almost zero methylation in the nucleosomal regions (Fig. 2*A*). This result confirms the correct assembly of the dinucleosomes, and it validates that the nucleosomes remained intact throughout the methylation experiment. For comparison of the different experiments, linker methylation levels were normalized for each experiment (Fig. 2*B*). Comparison of the normalized methylation data revealed that the methylation levels of equivalent CpG sites in the three different linkers were overall similar. However, there were specific differences of up to ±30% (Fig. 2*C*) many of which were significant when considering the technical fluctuations between the repeated experiments and applying a multiple testing correction (Fig. 2*D*). Of note, when comparing the 70 and 58 bp linkers, the strong and significant methylation differences were not clustered at the CpG sites at or near to the 12 bp deletions in the 58 bp linker substrates, but spread over the entire linker regions. In the case of Linker-58(1), the CpG sites 1 to 3 of Linker-70 were removed which brought the sites 4 and 5 into the direct contact of nucleosome 1. Nevertheless, the largest methylation differences were observed at CpG sites 4, 5, 6, 9, and 11 where the strongest effects were observed. In Linker-58(2), the CpG sites 6 to 8 in the middle of Linker-70 were removed such that no direct nucleosomal contacts were altered. Nevertheless, large methylation differences were observed throughout the linker, with large changes at CpG sites 5, 9, 12, 13, and strongest effects at site 11. Of note, the direct ±2 bp flanking sequences of the equivalent CpG sites which are most relevant for DNMT3A activity (14, 17) were not affected by the 12 bp deletions at any of the CpG sites, meaning that flanking sequence preference effects cannot explain the divergence in methylation levels.

**Figure 3:**
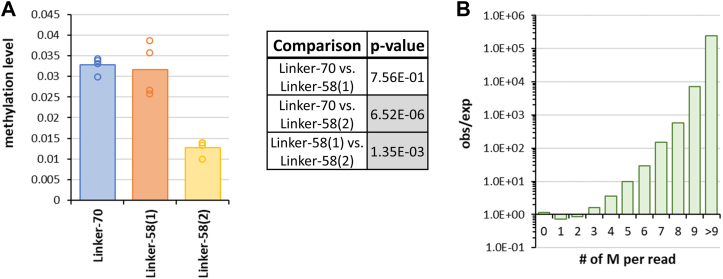
**DNMT3AC/3B3C methylation of linker CpG sites determined by competitive methylation of all three dinucleosome substrates.***A*, average linker methylation levels of all three dinucleosomes, methylated in competition in one reaction tube. Exemplary methylation profiles are shown in Figure S4. The table provides *p* values for the pairwise comparison of the methylation levels determined by two-sided *t* test with equal variance. Significant *p* values (*p* < 0.05/3, considering multiple testing) are shaded in *gray*. *B*, enrichment of sequence reads with many methylation events in the entire data set. The distribution of the total number of methylation events per sequence read was determined. Based on the average methylation level of the pool, an expected number of sequence reads with each number of methyl groups was calculated by binomial statistics and the observed/expected ratio (obs/exp) determined.

In order to compare the rates of methylation of all three dinucleosome substrates, they were all mixed and methylated by DNMT3AC/3B3C in a competitive reaction and methylation of the individual substrates determined by bisulfite sequencing. In all substrates, the nucleosomal regions were protected from methylation, indicating that the nucleosomes were intact throughout the experiments (Fig. S4). However, under these conditions, overall methylation levels were lower than in the previous reactions, because the ratio of nucleosomal substrates to enzyme and the methylation time had been reduced. This allowed a reliable determination of the methylation rates on the basis of the turnover levels, because no saturation effects had to be considered. As shown in Figure 3*A*, the Linker-70 and Linker-58(1) nucleosomes were methylated equally, while Linker-58(2) was methylated at a significantly reduced rate. The 60% reduced methylation of Linker-58(2) cannot be explained by a reduced preference of DNMT3A for its CpG sites, because the average ± 2 bp flanking sequence preferences of the CpG sites of Linker-70, Linker-58(1) and Linker-58(2) are identical within ±5%, also pointing toward more complex effects triggered by the nucleosome context.

Finally, as this data set provides readout of DNA methylation at low overall methylation levels, it was used to determine the distribution of methylation events on the individual sequencing reads which directly represents the distribution of methylation events on the individual product DNA molecules. For this, all methylation reactions were pooled and the number of reads observed with i methylation events was determined for i = 0 up to i > 9. For each methylation level, the expected number of sequence reads containing this number of methyl groups was determined based on the overall methylation level of the pool and the overall number of reads using a binomial distribution. Based on this, the observed/expected value for the number of reads with i methyl groups was calculated. As shown in Figure 3*B*, a very strong overrepresentation of sequencing reads with high numbers of methylation events was observed. Due to the high number of reads, all overrepresentations starting from i = 3 were highly significant with *p* values > 10^99^ (based on binomial distributions).

In summary, strong effects on nucleosome dependent linker CpG site methylation levels were observed after removal of parts of the linker DNA at sites far away from the sites of the deletions. Moreover, methylation levels of individual product molecules were not statistically distributed, but molecules with a high number of methylation events were highly overrepresented. These findings will be interpreted in the discussion chapter.

### Comparison of methylation patterns generated on free DNA and nucleosomal DNA

As described above, in the cryo-EM structure of the DNMT3A2/3B3-nucleosome complex, one of the terminal DNMT3B3 subunits forms a direct contact to the nucleosome acidic patch (27). To investigate the mechanistic role of this contact, we aimed to measure the recruitment of DNMT3A complexes to nucleosomal substrates. To address the direct effect of the DNMT3B3-acidic patch contact, we mutated the two critical arginine residues of the contact in DNMT3B3C by charge reversal creating the R740E/R743E double mutant (RE mutant). These amino acid exchanges had been investigated previously and the individual mutations were shown to lead to a reduction of nucleosome core complex binding of DNMT3A2/DNMT3B3 complexes (27). Of note, in the heterotetrameric DNMT3AC/3B3C complex, only the inner DNMT3AC subunits are catalytically active, while the outer DNMT3B3C subunits mediate the nucleosome contact. Hence, mutation of the acidic patch contact of DNMT3B3C allowed us to weaken specifically the acidic patch contact of the tetramer without directly affecting the catalytic activity of its DNMT3AC subunits.

**Figure 4:**
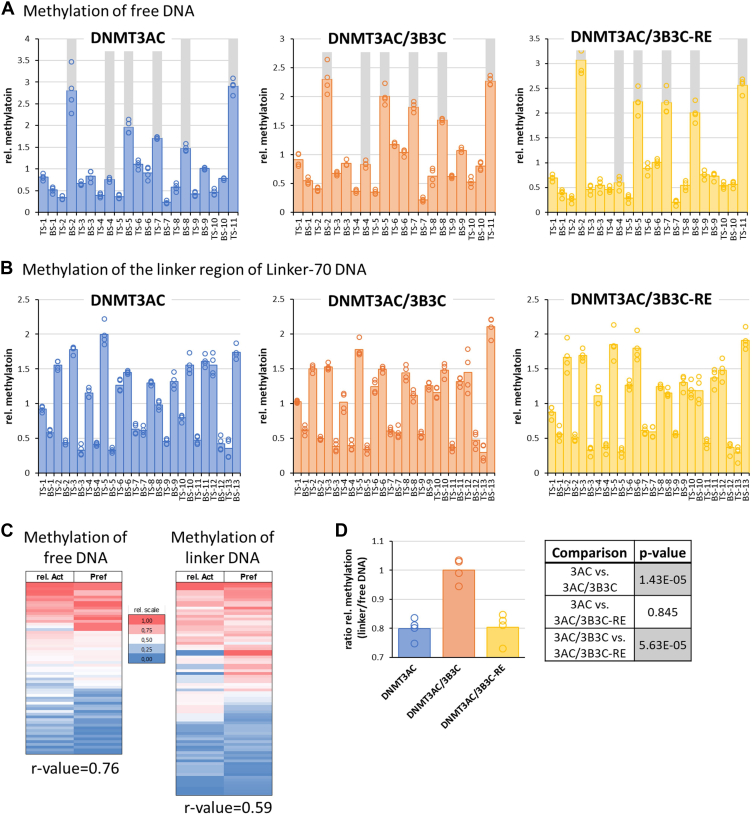
**Results of the competitive methylation of dinucleosomes linker regions and free DNA by DNMT3AC, DNMT3AC/3B3C, and DNMT3AC/3B3C-RE mutant.***A*, relative CpG site methylation levels of free DNA determined in four independent competitive methylation experiments. The sequence and CpG site annotation of the free DNA is provided in Figure S5*A*. The 6 most preferred sites based on DNMT3A flanking sequence preferences literature data (17) are shaded in *gray*. *B*, relative methylation levels of linker DNA in the Linker-70 dinucleosomes substrate determined in the four independent competitive methylation experiments. *C*, heatmap comparing the relative DNMT3AC/3B3C activity (rel. Act.) on free DNA and Linker-70 linker DNA with the flanking sequence preferences of the corresponding CpG sites (Pref) sorted by the average of both columns after scaling. Pearson r-values are indicated below. The scatter plots showing the correlations of the heatmaps are provided in Figure S5, *B* and *C*. *D*, ratio of the average methylation activities of DNMT3AC, DNMT3AC/3B3C, and DNMT3AC/3B3C-RE on dinucleosomes and free DNA. The table provides the *p* values of pairwise comparisons based on two-sided *t* test with equal variance. Significant *p* values (*p* < 0.05/3, considering multiple testing) are shaded in *gray*.

To compare the recruitment of DNMT3AC/3B3C and DNMT3AC/3B3C-RE heterotetramers to nucleosomal substrates and linker DNA methylation directly, we conducted DNA methylation experiments in mixtures of nucleosomes and free DNA with a different sequence (Fig. S5*A*) allowing to analyze the DNA methylation of both substrates quantitatively by bisulfite sequencing. This competitive methylation approach allows to draw quantitative conclusions on the kinetic effects of nucleosomal recruitment of the DNMT3 enzymes, independent of potential variances in enzyme concentrations, purity and activity. Methylation of four independent experiments was analyzed, again showing very low levels of methylation in the nucleosomal part (Fig. S6). As first analysis, the methylation data of the free DNA and nucleosomal linker DNA were investigated separately. For this, each experiment was normalized to the average methylation observed on all CpG sites of the respective substrate revealing that the relative methylation levels observed in all four experimental repeats are highly correlated (Fig. 4, *A* and *B*). As expected and in agreement with previous data (6, 14, 17, 39, 40), the different DNMT3A complexes methylated the CpG sites in the free DNA with an about 20-fold ratio in methylation rates of the best and worst sites. To explore if these differences were determined by the flanking sequence preferences, we used the merged DNMT3A flanking sequence preference datasets of Gao *et al.* (2020) (14) and Dukatz *et al.* (2022) (17) to calculate NNCGNN preferences of DNMT3A which were then used to assign preferences to the CpG sites. Of note, strong methylation was observed at five sites, which indeed are among the six most preferred target sites based on the flanking sequences. One deviation between the expected and observed activities was observed at the bottom strand of site 4, which based on its CCCGCC sequence was expected to be strongly methylated, but this was not the case. This effect may be due to the larger sequence context of this site (GGCCCGCC/GGCGGGCC) which is very GC-rich and therefore likely to adopt unusual DNA conformations. Overall, the flanking sequence preferences of DNMT3A correlate very well with the observed DNMT3AC/3B3C activity at the corresponding sites yielding a Pearson r-value of 0.76 (Fig. 4*C*). In conclusion, the free DNA is methylated largely following the flanking sequence preferences of DNMT3A in this experiment.

The methylation data of the linker DNA (Fig. 4*B*) showed a 10-fold ratio in methylation rates of the best and worst sites, but strikingly the correlation of the DNMT3AC/3B3 CpG site activity with the corresponding flanking sequence preferences of DNMT3A only resulted in a Pearson r-value = 0.59 (Fig. 4*C*). Hence, the correlation of DNMT3A flanking sequence preferences and CpG site activities of DNTM3AC/3B3C on linker DNA was significantly weaker than the correlation on naked DNA (*p* value = 0.034 for the comparison of both correlations based on Fisher's z-transformation). This finding indicates that the dinucleosome context influences the activity of the DNMT3AC/3B3C tetramers, and this effect weakens the influence of the flanking sequence on the enzymatic activity.

### Biochemical effects of the DNMT3B3-acidic patch contact

With all three enzymes, we observed a reduced methylation activity on the linker DNA when compared to free DNA (Fig. S6). This finding can be explained by the shielding of linker DNA by the positively charged histone tails (33, 41). To compare the methylation of the linker DNA with the free DNA quantitatively, we used the five most highly methylated sites on the free DNA as reference sites for free DNA methylation. Then, we determined the activity ratio for methylation of the Linker-70 dinucleosome linker sites divided by activity on free DNA in the individual competitive methylation experiments conducted with the three different DNMT3A tetramers (Fig. 4*D*). This analysis revealed that the relative methylation of nucleosome linker DNA was about 20% higher with WT DNMT3AC/3B3C than with the corresponding RE mutant or with DNMT3A with highly significant *p* values in both comparisons. This result indicates that the nucleosomal recruitment of DNMT3AC/3B3C *via* the acidic patch contact improves the methylation of the linker DNA. Relative linker DNA methylation of DNMT3AC was as low as the RE mutant indicating that the acidic patch contact of DNMT3AC is weaker than that of DNMT3B3C as proposed previously based on structural modeling (27).

### Effects of nucleosome recruitment on DNMT3A activity

**Figure 5:**
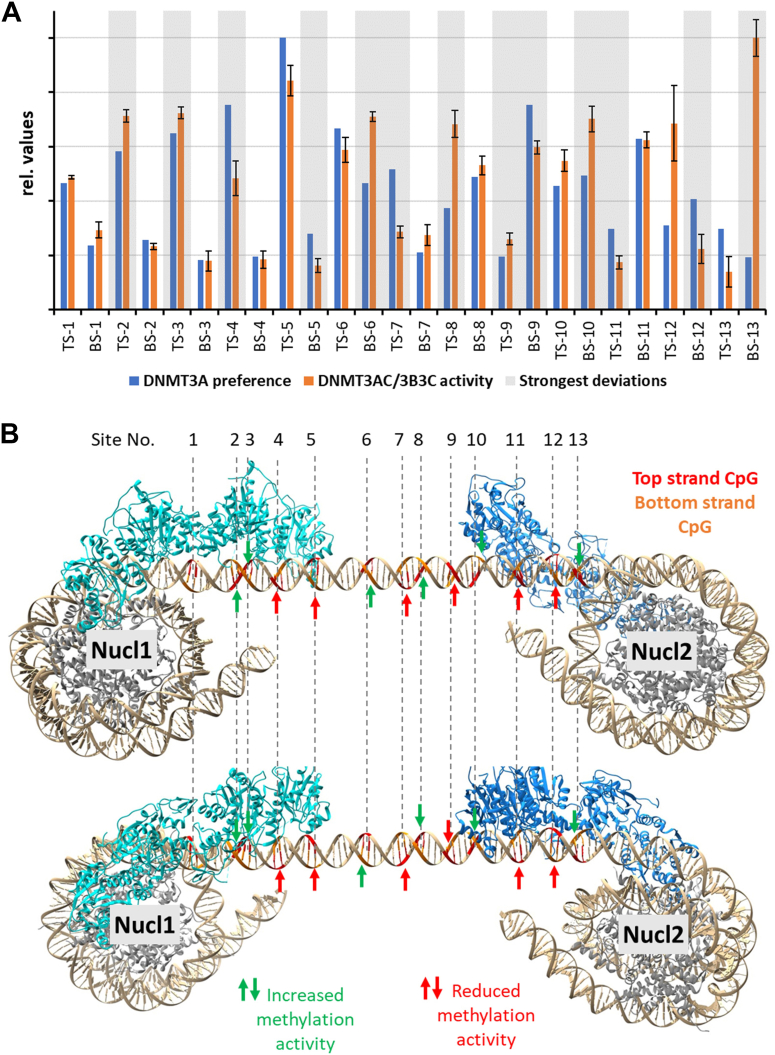
**Effects of the recruitment of DNMT3AC/3B3C to the nucleosome.***A*, comparison of the flanking sequence preferences of DNMT3A and the relative DNMT3AC/3B3C activity at all linker CpG sites. The error bars show the standard deviation. Individual data points are provided in Figure S7. Thirteen out of the 26 sites with significant difference and deviations > 10% are shaded *gray*. *p*-values were determined by Z-statistics and *p* < 0.05/26 (considering multiple testing) was indicated as significant. *B*, Visualization of the sites showing the strongest nucleosomal effects on methylation rates on a dinucleosome model generated by rigid body superposition as described in the Methods section. *Top* strand CpG (in the notation of this study) are colored *red*, *bottom* strand CpG *orange*. The position of sites with most prominent nucleosome dependent stimulation or repression of activity are highlighted by *green* (increased activity) or *red arrows* (reduced activity). The two images show the same model rotated by approximately 90° about the linker DNA axis.

Next, we were interested to elucidate the consequences of the nucleosomal targeting of DNMT3AC/3B3C complexes on the linker DNA methylation rates in more details. For this, we compared the normalized DNMT3A flanking sequence preferences and the experimentally observed DNMT3AC/3B3C methylation activities on the linker DNA of the Linker-70 dinucleosome. We argue that deviations between the expected and observed methylation activities can be related to the specific vertical and rotational placement of the DNMT3AC/3B3C tetramers on the linker DNA which is enforced by the nucleosome binding and will be referred to as nucleosomal effect on CpG site methylation activities from now on. To identify these nucleosome dependent activity changes, we first selected the sites with the largest deviations between observed and expected methylation rates (Fig. 5*A*). Then, we highlighted these positions in a dinucleosome model and indicated if they were hypermethylated or hypomethylated when compared with the expected methylation based on the flanking sequence preferences (Fig. 5*B*). This analysis clearly illustrates the guidance of the methylation by the nucleosome targeting for the CpG sites nearby the nucleosome core regions (sites 1–5 and 10–13), because CpG sites oriented toward the DNMT3A/3B3 complexes are methylated better than expected based on their flanking sequence, while sites with a rotational placement on the DNA pointing away from the enzyme are methylated worse than expected. This finding is in agreement with the conjecture that nucleosomal anchorage occurs on both sides of the linker DNA and it reduces the inherent flexibility of the enzyme-DNA complex, for example by preventing the rotation of the DNMT3A complex about the DNA axis or sliding of DNMT3A complexes along the DNA axis. However, strong deviations between observed and expected methylation rates were also noticed at the CpG sites 6 to 9 which are not in close contact with any of the nucleosomes indicating that nucleosome binding also affects methylation activities at these distant CpGs “though space”, a question that will be taken up in the discussion chapter.

## Discussion

It was discovered in 2020 that DNMT3A/3B3 heterotetramers directly bind to nucleosomes *via* a contact of the distal DNMT3B3 subunit to the H2A/H2B acidic patch on the surface of the histone octamer (27). In the same work, this contact was shown to affect nucleosome interaction and chromatin binding of DNMT3A/3B3 (27). Later kinetic studies demonstrated that the methylation levels of two characteristic CpG sites in the linker DNA next to the nucleosome agree very well with the DNMT3A/3B3-nucleosome structure, because one CpG site ideally positioned to approach an active site of the complex was methylated very fast while another site not able to interact with an active site was poorly methylated (33). However, the detailed effects of the acidic patch contact on the kinetics of linker DNA methylation rates remained unresolved. In this study, we determined the methylation activities of CpG sites on free DNA and dinucleosome substrates by DNMT3AC/3B3C and isolated DNMT3AC complexes. DNMT3AC/3B3C-RE mutant complexes with a mutated acidic patch contact were used as control. Although CpG site-specific methylation activities observed on free DNA basically followed the known flanking sequence preferences of DNMT3A (6, 14, 17), dinucleosomal linker DNA was methylated with different preferences. In all our experiments, we observed strong nucleosomal effects on methylation activities on both sides of the linker DNA, suggesting that both nucleosomes are contacted by a DNMT3 complex. However, we also observed strong nucleosomal effects in the middle of the 70 bp linker DNA, far away from both nucleosomes. Moreover, when comparing methylation patterns observed on the 70 bp and on two different 58 bp linker dinucleosomes, we discovered changes of the CpG site methylation activities at sites distant to the 12 bp deletions in the linker DNA sequence. These observations indicate that nucleosomal effect can act “through space”, a finding that cannot be explained by the recruitment of one DNMT3 complex at each nucleosome alone.

**Figure 6:**
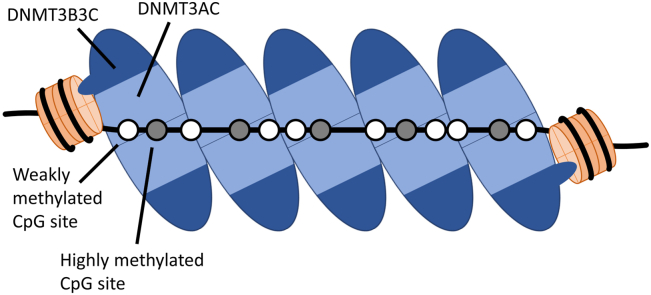
**Schematic picture of the multimerization of DNMT3AC/3B3C complex heterotetramers on dinucleosomes.** CpG cytosine residues that can easily reach a DNMT3A active site after base flipping are methylated more efficiently than expected by their flanking sequence (symbolized by *gray* shading), CpG cytosine residues placed such that they cannot bind to an active site are methylated only weakly (symbolized by the *white* color). The DNMT3 complexes and nucleosome are not drawn to scale.

Several mechanisms are conceivable how DNMT3 complexes could interact with dinucleosomes and mediate linker DNA methylation. Complexes could be transiently anchored at the nucleosomes but later released and move along the linker DNA. Alternatively, DNMT3 complexes could stay anchored on the nucleosomes, but additional, freely moving complexes could bind and methylate the linker DNA. However, both these models are not in agreement with our data, because we observe nucleosomal effects on the methylation activity along the entire linker region, but enzyme complexes not contacting the nucleosome should act on the linker DNA with the same sequence preferences as on free DNA. The only model explaining our biochemical data is that DNMT3 complexes multimerize on the linker DNA, similarly as observed previously on free DNA (21, 22, 28). In this model (Fig. 6), the enzyme multimers are anchored on both sides on the nucleosomes and the DNMT3 complexes in between are vertically and rotationally positioned on the DNA depending on the linker DNA length and potentially also the linker DNA sequence. This multimerization spatially organizes the complexes, aligning active site pockets of DNMT3A complexes with the position of individual cytosine residues in CpG sites. Depending on this arrangement, some CpG cytosine residues can easily reach a nearby active site by base flipping. These sites are the ones that are methylated faster than expected on the basis of their flanking sequence (Figs.5B and 6). Other CpG cytosines cannot reach an active site even after base flipping, which are the sites that are methylated less than expected based on their flanking sequence. This multimerization model can also explain the reduced methylation of the Linker-58(2) dinucleosome which was observed although the average preferences for the CpG flanking sites in all three dinucleosome substrates are very similar, because due to the specific deletion in the sequence, the relative arrangement of CpG sites and bound DNMT3A complexes are changed. In the case of the Linker-58(2) dinucleosome substrate, this apparently led to a less favorable overall fit between the positioning of the CpG sites and DNMT3A active centers in the DNMT3-DNA fiber. Moreover, for the given average methylation levels, we observed very strong overrepresentations of product DNA molecules with high methylation states, which further supports the conclusion that the DNMT3AC complexes form protein fibers on the DNA in a cooperative reaction. A hypothetical processive reaction mechanism cannot explain these findings, because in a processive reaction, the methylation rate of a CpG site is determined by its flanking sequence preference, but not by its placement on the linker DNA with respect to the nucleosomes as observed here. It will be interesting to investigate the interaction of DNMT3A with dinucleosomes directly in structural cryo-EM studies and dynamically in molecular dynamics simulations with atomic resolution in future work.

We also investigated the direct effect of the DNMT3-acidic patch contact on the activity of DNMT3 complexes with competitive methylation experiments of dinucleosomes and free DNA. Our data show that the acidic patch contact improves nucleosomal recruitment and linker DNA methylation by DNMT3A/3B3 complexes suggesting that this effect could also contribute to cellular DNA methylation. So far, it is not known if DNMT3A can also contact the acidic patch. High sequence homology of the amino acid region involved in the acidic patch contact between DNMT3A and DNMT3B3 (33), as well as the similar methylation patterns on linker DNA observed with DNMT3AC/3B3C and isolated DNMT3AC complexes reported here and also previously (33) suggest that this interaction might exist. On the other hand, based on structural modeling it had been proposed that the acidic patch contact of DNMT3A is sterically precluded (27). Our kinetic data indeed suggest that the acidic patch contact of DNMT3A is weaker than that of DNMT3B3. The different strength of the acidic patch contacts of DNMT3A and DNMT3B3 may explain the specific regulatory roles of DNMT3B3 in cells (12, 42, 43, 44). Unfortunately, the kinetic effects of a potential acidic patch interaction of DNMT3A cannot be investigated by mutation of the corresponding arginine residues (R882 and R885 in human DNMT3A), because both residues have very important mechanistic roles on their own. R885 is a key residue in the RD interface and the R885A mutation has been shown to destroy this interface and abrogate catalytic activity (21, 45). R882 is frequently mutated in acute myeloid leukemia patients (46). Mutations of this residue affect the DNMT3A RD interface and flanking sequence preferences (6, 23, 40, 47), which would obscure potential effect of the acidic patch contact. It has not been investigated so far, if effects on the nucleosome interaction of DNMT3A contribute to the pathogenicity of R882H mutations in AML.

To describe the principle arrangement of DNMT3A multimers on linker DNA, we resort to previous work that has shown DNMT3A co-methylation of CpG sites in the same DNA strand when they are separated by 12 to 13 bps (16). This setup corresponds to a distance of 14 to 15 bps between the target cytosine residues. Hence, there is space for about 5 DNMT3AC/3B3C complexes on the 70 bp linker DNA and about 4 complexes on the 58 bp linkers, suggesting that 3 or 2 DNMT3A complexes are recruited to the linker DNA in between the two complexes anchored to the nucleosomes at both sides. Multimerization of DNMT3A complexes on linker DNA is an interesting observation that could influence DNA methylation patterns in cells depending on nucleosome positioning. However, conversely our data also suggest that the binding preferences of DNMT3A and consequent arrangement of DNMT3A complexes in the protein/DNA fiber could contribute to nucleosome positioning by pushing nucleosomes outward or pulling them inward. It will be interesting in future work to investigate these hypotheses by additional experiments comparing nucleosome positioning and DNA methylation patterns, ideally in cell lines only containing DNMT3A as an active DNA methyltransferase. Moreover, DNMT3A has been shown to bind strongly to heterochromatic regions and chromatin (21, 48) and multimerization on DNA (22), and the acidic patch contact (27) has been shown to participate in this. Hence, it is plausible to postulate that multimerization of DNMT3A on linker DNA occurs in heterochromatin and it may particularly contribute to methylation patterns and nucleosome positioning in these regions.

In the cellular context, it must be considered that the chromatin interaction of DNMT3A complexes is multivalent not only based on direct DNA binding and nucleosome acidic patch interaction both mediated by the catalytic domain and investigated in this work. In addition, other domains of full-length DNMT3A also interact with different histone post-translational modifications (PTMs) including H3K4me0, H3K36me2/3, and H2AK119ub1 (10). These domains are in flexible arrangements able to directly regulate the catalytic activity of DNMT3A depending on their histone-PTM interaction (49, 50). Futures work will be required to integrate our understanding about the combinatorial roles of all these interactions and their combined effect on the regulation of DNMT3A activity in cells.

## Experimental procedures

### Purification of His-DNMT3AC

The His-tagged catalytic domain (amino acid 608–908) of murine DNMT3A was recombinantly expressed in *E. coli* cells and purified as described (17, 33). Briefly, DNMT3AC was overexpressed in BL21 (DE3) codon + RIL *E. coli* cells grown in LB medium until an *A*_600_ of 0.6 was reached. The DNMT3AC expression was induced for 14 h at 20 °C by the addition of 0.5 mM isopropyl-β-D-1-thiogalactopyranoside. The cells were harvested by centrifugation at 4500 rcf for 20 min. The cell pellet was resuspended in sonication buffer (30 mM KH_2_PO_4_/K_2_HPO_4_ pH 7.5, 500 mM KCl, 1 mM EDTA, 0.2 mM DTT, 10% glycerol, and 20 mM imidazole), and the cells were disrupted by sonication (25 cycles with 15 s active sonication and 30 s pause, 35% amplitude, SONOPLUS ultrasonic homogenizer, Bandelin). The cell lysate was centrifuged at 40,000 rcf for 60 min and the supernatant was loaded onto a nickel-nitrilotriacetic acid-agarose matrix column which was equilibrated with sonication buffer. After washing with 100 ml of sonication buffer, the protein was eluted with sonication buffer containing 220 mM imidazole. Fractions were collected, pooled according to yield and purity. The purified protein was dialyzed against 20 mM Hepes pH 7.5, 200 mM KCl, 0.2 mM DTT, 1 mM EDTA, and 10% glycerol, flash-frozen in liquid nitrogen and stored at −80 °C. Protein purity was determined using Coomassie brilliant blue (BB)-stained 12% SDS polyacrylamide gels (Fig. S2*B*).

### Cloning and purification of DNMT3AC/3B3C tetrameric complexes

The DNMT3B3C R740E/R743E double mutant was generated by site-directed mutagenesis using the primers listed in Table S1. The purification of DNMT3AC/3B3C and DNMT3AC/3B3C-RE tetrameric complexes was performed by double affinity purification basically as described in (37) (Fig. S2*A*). In short, MBP-TEV-DNMT3AC (amino acid 608–908) and His-DNMT3B3C (or its RE mutant) (amino acid 592–898) were co-overexpressed in BL21 (DE3) codon + RIL *E. coli* cells. In the following, the TEV site in MBP-TEV-DNMT3AC will only be indicated if this is relevant. The cells were grown in LB medium until an *A*_600_ of 0.6 was reached. The protein expression was induced for 14 h at 20 °C by the addition of 0.5 mM isopropyl-β-D-1-thiogalactopyranoside. The cells were harvested by centrifugation at 4500 rcf for 20 min. The cell pellet was resuspended in sonication buffer (30 mM KH_2_PO_4_/K_2_HPO_4_ pH 7.5, 500 mM KCl, 1 mM EDTA, 0.2 mM DTT, and 10% glycerol), and the cells were disrupted by sonication (25 cycles with 15 s active sonication and 30 s pause, 35% amplitude, SONOPLUS ultrasonic homogenizer, Bandelin) The heterotetrameric complexes were purified first over an amylose matrix for binding of MBP-tag containing protein complexes. After washing with 100 ml of sonication buffer, the protein was eluted with sonication buffer containing 20 mM maltose. The eluate was then applied to a second purification step using a nickel-nitrilotriacetic acid-agarose matrix capturing all protein complexes also containing a His-tagged subunit (Fig. S2*A*). After washing with 100 ml of sonication buffer (30 mM KH_2_PO_4_/K_2_HPO_4_ pH 7.5, 500 mM KCl, 1 mM EDTA, 0.2 mM DTT, 10% glycerol, and 20 mM imidazole), the protein was eluted with sonication buffer containing 220 mM imidazole. Fractions were collected, pooled according to yield and purity. The resulting purified heterotetrameric complexes were dialyzed against 20 mM Hepes pH 7.5, 200 mM KCl, 0.2 mM DTT, 1 mM EDTA, and 10% glycerol, flash-frozen in liquid nitrogen and stored at −80 °C. Protein purity was determined using Coomassie BB-stained 12% SDS polyacrylamide gels (Fig. S2*B*). Prior to the methylation reactions, the MBP-tag was cleaved off by treatment with 35 μM TEV protease for 15 min at 20 °C in 20 mM Hepes pH 7.5, 200 mM KCl, 0.2 mM DTT, 1 mM EDTA, and 10% glycerol which fully restores the catalytic activity of DNMT3AC as described (47). Complete TEV cleavage of the MBP-tagged DNMT3AC fusion protein under our conditions is shown in Figure S2*C*. We have shown in our previous work, that the TEV protease does not digest nucleosomes (33). TEV protease was purified from bacterial expression plasmid pRK793 (Addgene #8827) as described (51).

To measure the long-term stability of the purified DNMT3AC/3B3C complexes, the binding of MBP-DNMT3AC/His-DNMT3B3C heterotetramers to amylose beads was analyzed after 12 months of storage (Fig. S2*D*). His-DNMT3AC and MBP-DNMT3AC were used as controls. The proteins were bound to amylose beads and washed with interaction buffer (25 mM Tris/HCl pH 7.5, 100 mM KCl, 5 mM MgCl_2_, 10% glycerol, 0.1% IGEPAL, and 200 μM phenylmethylsulfonyl fluoride). Flow-through and wash were combined, the proteins precipitated by trichloroacetic acid and dissolved in SDS gel loading buffer. The protein bound to the beads was eluted by heating to 95° for 10 min in SDS gel loading buffer. Afterward, all samples were separated on SDS-PAGE and stained with Coomassie BB. Exchange of MBP and His-tagged subunits of MBP-DNMT3AC/His-DNMT3B3C complexes should lead to elevated levels of MBP-DNMT3AC in the eluate and increased levels of His-DNMT3B3C in the flow-through.

### Generation of histone octamers

Purification of individual histone proteins was performed as described (33, 52). The reconstitution of histone octamers was carried out basically as described (33, 53). First, individual histone proteins were dissolved in unfolding buffer (20 mM Tris/HCl pH 7.5, 7 M guanidinium chloride, 5 mM DTT), and their concentrations were determined spectrophotometrically. The proteins were then mixed at a 1:1.2 ratio (H3/H4 to H2A/H2B) and dialyzed overnight against two changes of refolding buffer (10 mM Tris/HCl pH 7.5, 1 mM EDTA, 2 M NaCl, and 5 mM β-mercaptoethanol). To isolate the octamers from substoichiometric assemblies, the sample was separated by size-exclusion chromatography using a Superdex 200 16/60 PG column equilibrated in refolding buffer (Fig. S1*A*). The collected fractions of the octamer peak were pooled based on purity and concentrated tenfold using Amicon Ultra-4 centrifugal filters (30 kDa cutoff, Merck Millipore). Finally, the histone octamer samples were flash-frozen in liquid nitrogen and stored at −80 °C.

### Nucleosome reconstitution

To generate recombinant dinucleosomes, DNA templates containing two Widom 601 nucleosome binding sites (35) were prepared by TOPO-TA cloning and Gibson Assembly. Primers used for the generation of the dinucleosome DNAs are listed in Table S2. In the first step, a silent *MluI* restriction site was introduced into the 601 sequences, followed by the creation of two distinguishable versions of the Widom 601 nucleosome binding sites (Nuc1 and Nuc2) *via* site-directed mutagenesis and TOPO-TA cloning. Subsequently, a linker was introduced into the Nuc2-containing plasmid and the Nuc1 and Nuc2 sequences, together with the linker, were combined into one construct by Gibson Assembly. Finally, the 2 nucleosome binding sites were separated by either a linker of 70 bps with 13 CpG sites (Linker-70) or two different versions of linkers with 58 bps, Linker-58(1) and Linker-58(2), each containing 10 CpGs. For large-scale production of the dinucleosome DNA template, preparative PCR amplification was performed. The resulting DNA was purified and then combined with a twofold excess of histone octamers. Samples were dialyzed in Slide-A-Lyzer microdialysis devices (Thermo Fisher Scientific) against a high-salt buffer (10 mM Tris/HCl pH 7.5, 2 M NaCl, 1 mM EDTA, 1 mM DTT). Over the course of 24 h, the high-salt buffer was gradually replaced with 2 L of low-salt buffer (10 mM Tris/HCl pH 7.5, 250 mM NaCl, 1 mM EDTA, 1 mM DTT) to facilitate nucleosome assembly. Subsequently, the samples were dialyzed overnight against storage buffer (10 mM Tris/HCl pH 7.5, 1 mM EDTA, 1 mM DTT, 20% glycerol), flash-frozen in liquid nitrogen, and stored at −80 °C. Dinucleosome reconstitution was validated using gel retardation (Fig. S1*B*) and restriction digestion (Fig. S1*C*) assays following the approach described in our previous work (33).

### DNA methylation experiments

For the methylation experiments with different dinucleosome substrates, each dinucleosome variant was digested with MluI-HF (NEB) for 15 min at 37 °C in 7 μl NEB Cutsmart buffer (50 mM KOAc/20 mM Tris-acetate pH 7.9, 10 mM magnesium acetate, 100 μg/ml bovine serum albumin). This enzyme cleaves the DNA at sites that are protected by the nucleosome. Hence, residual free DNA and DNA incorporated into mononucleosomes is cut and cannot be amplified in the PCR reactions after bisulfite conversion, efficiently excluding these molecules from the analysis. Finally, 0.1 pmol of each dinucleosome variant were used in the methylation reactions. For the competitive methylation experiments of dinucleosome substrate with free DNA, 0.1 pmol free CpG-rich DNA (162 bps, 11 CpG sites) was added. Of note, the free DNA spiked into the competitive methylation assays does not contain an MluI cleavage site. For triple competitive reactions of different dinucleosomes, 0.033 pmol of each dinucleosome variant were mixed together after MluI-HF digestion and used as a substrate for the methylation reaction.

For the methylation reactions, DNMT3AC (0.8 μM monomers) or TEV-cleaved DNMT3AC/DNMT3B3C or its RE mutant (0.37 μM tetramers) was added in Cutsmart buffer supplemented with 4 mM EDTA and 1 μM AdoMet (Sigma-Aldrich). The methylation reactions were performed in a volume of 20 μl for up to 2 h at 37 °C. To stop the reactions at different time points, samples were taken and flash-frozen in liquid nitrogen. To remove all nucleosome-bound proteins, proteinase K was added to the reaction, and the sample was incubated for 60 min at 37 °C. The resulting DNA was purified from the reaction mixture using the Nucleospin Gel and PCR cleanup kit (Macherey-Nagel). Bisulfite conversion of the DNA was performed using the EZ DNA Methylation-Lightning kit (Zymo Research). During data analysis, methylation levels representing technical repeats (*i.e.* the sequencing results of linker DNA methylation read from the direction of nucleosome 1 or nucleosome 2) were averaged. Data from experimental replicates were normalized as described in the results section, most time to the average methylation levels of the corresponding substrate in the respective experimental repeat. These values were referred to as relative methylation levels.

### Library preparation and sequencing analysis

DNA libraries for Illumina NGS were prepared with a two-step PCR approach, as similarly described (38). For this, the Nucleosome 1–Linker–Nucleosome 2 sequence was split into two amplicons, one comprising the Nucleosome 1–Linker and the second comprising Linker–Nucleosome 2. For library preparation, 1 μl of bisulfite-converted DNA was amplified in a first PCR reaction (15 cycles) using barcoded primers and HotStartTaq DNA Polymerase (QIAGEN). In the second PCR reaction (15 cycles), 1 μl of PCR1 product was amplified using i5 and i7 indexing primers and Q5 polymerase (New England Biolabs). Successful amplification was verified by agarose gel electrophoresis. Samples were pooled in equimolar amounts, purified with NucleoSpin Gel and PCR Clean-up kit and used for Illumina paired end 2 × 250 bp sequencing conducted at Novogene. Bioinformatic analysis of NGS data was conducted using a local instance of the Galaxy server (54). Sequencing primers are provided in Table S3. Obtained sequence reads were trimmed with the Trim Galore! Tool (github.com/FelixKrueger/TrimGalore), discarding tails with a quality score below 20. Afterward reads were paired using PEAR (55). Reads were filtered according to the expected DNA length using the Galaxy Filter FASTQ tool (56). The demultiplexing was done by the selection of the reads with specific combinations of barcodes and Illumina adapters which were then mapped against the corresponding reference sequence using bwameth (github.com/brentp/bwa-meth). In case of triple competitive methylated dinucleosome variants (Linker-70, 58(1), 58(2)), an additional filtering step for the internal barcode of the linker region was implemented in the demultiplexing. Finally, the DNA methylation of individual CpG sites was computed using MethylDackel (github.com/dpryan79/MethylDackel).

### Generation of the dinucleosomes model with bound DNMT3A/3B3

To create a dinucleosome model with bound DNMT3A/3B3, the DNA molecules of two copies of the DNMT3A2/3B3 nucleosome complexes (pdb 6PA7) were connected with a synthetic 70 bp linker DNA in B-DNA conformation in Chimera 1.19 (57). In this model, nucleosome 2 contains the structurally resolved DNMT3A2/3B3 complex contacting the linker DNA. In nucleosome 1, the DNMT3A2/3B3 complex was placed at the symmetrically related acidic patch of the other surface of the octamer by superposition of the corresponding H2A/H2B subunits and manual adjustment to relieve steric clashes with the linker DNA. Of note, this model has been created by rigid body superposition without energy minimization not considering DNA flexibility and potential hinge movements of the DNMT3A2/3B3 complexes with respect to the nucleosome. It has only been used for the visual illustration of the data.

### Statistics and reproducibility

Pearson correlation factors (r-values) were determined with the Excel Correl function. The significance of correlations was tested by T-statistics. The *p* values of comparisons of data sets were determined by two-sided *t* test assuming equal variance with the Excel Ttest function. The number of experimental repeats is indicated in each case. The significance of *p* values was assessed by including the Bonferroni multiple testing correction.

## Data availability

Analyzed raw sequencing data are available at https://doi.org/10.18419/DARUS-5490.

All other data are contained within the manuscript and its supporting information. The substrate DNA sequences are provided in Text S1-S4. The amino acid sequences of the enzymes are provided in Text S5-S8.

## Supporting information

This article contains supporting information.

## Conflict of interest

The authors declare that they have no conflicts of interest with the contents of this article.
