# Supplementary material for: Nucleosome linker DNA methylation by DNMT3A/DNMT3B3 is controlled by nucleosome binding and multimerization of DNMT3 complexes on DNA
Source: J Biol Chem. 2026 Jan 10;302(3):111154. doi: 10.1016/j.jbc.2026.111154 (PMC12876729; doi:10.1016/j.jbc.2026.111154)
Supplement: Supplement [file mmc1.pdf]

Nucleosome linker DNA methylation by DNMT3A/DNMT3B3 is controlled by nucleosome binding and multimerization of DNMT3 complexes on DNA

Nicole Gutekunst, Alexander Bröhm, Pavel Bashtrykov & Albert Jeltsch*

Supporting Information

Figure S1: Validation of nucleosome reconstitution.

Figure S2: Purification of the different DNMT3A proteins and complexes.

Figure S3: Quality control and processing steps of the nucleosome methylation experiments.

Figure S4: Exemplary data set showing the methylation levels in the top and bottom DNA strands determined in the competitive methylation of all three dinucleosomes substrates.

Figure S5: Additional information related to the competitive methylation of nucleosomal and free DNA by DNMT3AC/3B3C.

Figure S6: Exemplary data set showing the methylation levels in the top and bottom DNA strands determined in the competitive methylation of dinucleosomes substrates with free DNA.

Figure S7: Individual data points of the relative DNMT3AC/3B3C activity at all CpG sites shown in in the linker regions.

Text S1: DNA sequence of the dinucleosome substrate with Linker-70.

Text S2: DNA sequence of the dinucleosome substrate with Linker-58(1).

Text S3: DNA sequence of the dinucleosome substrate with Linker-58(2).

Text S4: DNA sequence of the CpG-rich DNA.

Text S5: Protein sequence of the His-DNMT3AC used in methylation assays.

Text S6: Protein sequence of the MBP-TEV-DNMT3AC used in methylation assays.

Text S7: Protein sequence of the His-DNMT3B3C used in methylation assays.

Text S8: Protein sequence of the His-DNMT3B3C-RE used in methylation assays.

Table S1: List of primers used for DNMT3B3 mutagenesis

Table S2: List of primers used for generation of the dinucleosome DNAs

Table S3: List of primers used for sequencing

Figure S1: Validation of nucleosome reconstitution. A) Size exclusion chromatogram of the histone mixture separated on a Superdex 200 16/60 PG column after histone octamer refolding. The octamer fraction was collected and used for the following experiments. **B)** Example of EMSA experiments showing dinucleosome reconstitution using the 70 bp linker and 58(1) and 58(2) linkers. Indicators mark the unshifted free DNA bands at 408 bp (70 bp linker) or 396 bp (58 bp linker) and the shifted dinucleosome bands. The images show 8% TB gels stained with GelRed. All lanes in the Linker-70 dinucleosome image were taken from the same original gel image. **C)** Example of restriction protection experiments showing Linker-70 dinucleosome reconstitution. Restriction digestion of free DNA and nucleosomal DNA with MluI and MfeI showing that free DNA is cleaved but nucleosomal bound DNA is protected from cleavage. The images show 8% TB gels stained with GelRed.

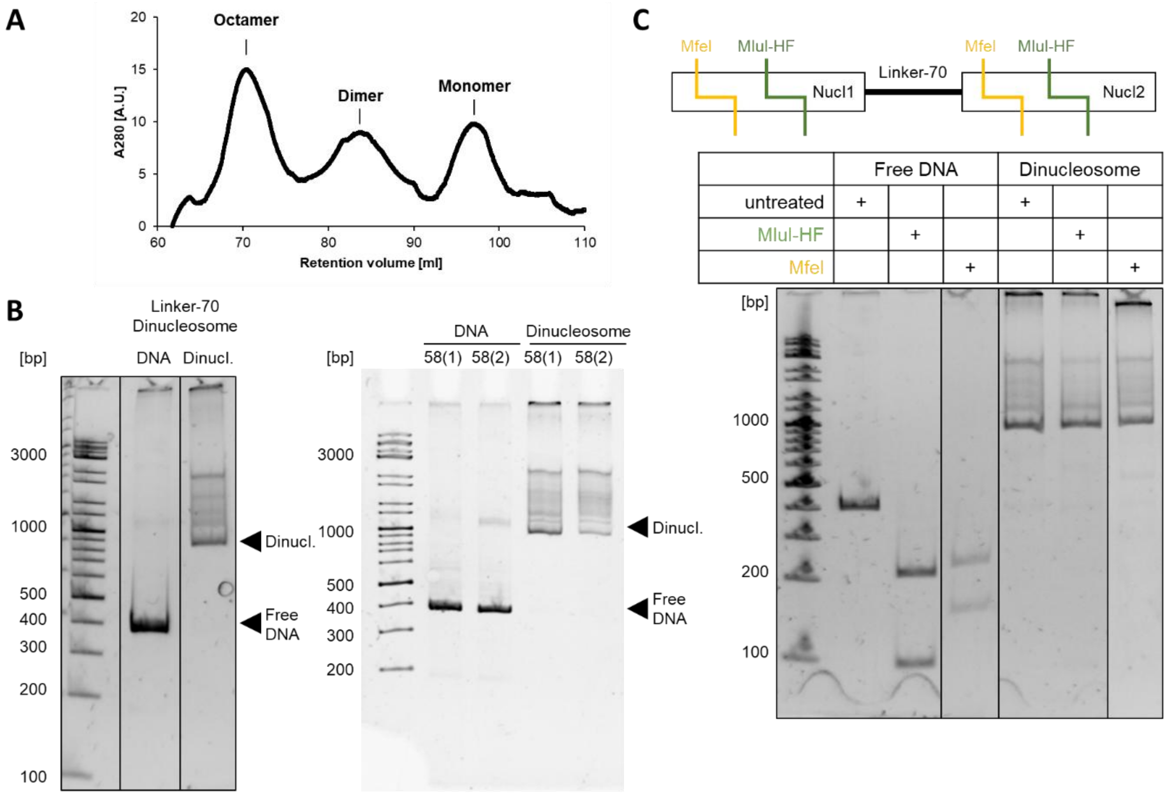

Figure S2: Purification of the different DNMT3A proteins and complexes. A) Scheme of the double tag protein purification method used for the purification of MBP-DNMT3AC/His-3B3C heterotetramers. **B)** Coomassie BB stained SDS-PAGE showing the purified DNMT3AC protein, DNMT3AC/3B3C and DNMT3AC/3B3C-RE complexes that were used in this study. The theoretical sizes of the proteins are MBD-TEV-DNMT3AC: 78.1 kDa, His-DNMT3AC: 36.8 kDa, His-DNMT3B3C: 29.5 kDa. **C)** Control experiment showing complete digestion of the MBP-TEV-DNMT3AC fusion protein into its MBP and DNMT3AC parts by TEV protease under our experimental conditions. The theoretical sizes of the proteins are: MBD-TEV-DNMT3AC: 78.1 kDa, cleaved MBP part: 40.3 kDa, cleaved DNMT3AC part: 34.5 kDa, MBP-TEV protease 71 kDa, His-DNMT3B3C: 29.5 kDa. **D)** Absence of subunit exchange in purified MBP-3AC/His-3B3C heterotetramers during storage. Purified MBP-3AC/His-3B3C was bound to amylose beads after 12 months of storage and the amounts of His- and MBP-subunits in the flow-through and eluate were determined. Purified His-3AC and MBP-3AC proteins were used as controls. Subunit exchange would lead to an enrichment of His-tagged subunits in the flow-through and enrichment of MBD-tagged subunits in the eluate. This is not detectable as indicated by the comparable flow-through to eluate ratio of MBP- and His-subunits of MBP-3AC/His-3B3C (lane 5 and 6) which is also matching that of the MPB-3AC control (lane 3 and 4). Moreover, the ratio of His- and MBP-subunits of MPB-3AC/His-3B3C is comparable in input (lane 9), flow-through (lane 5) and eluate (lane 6).

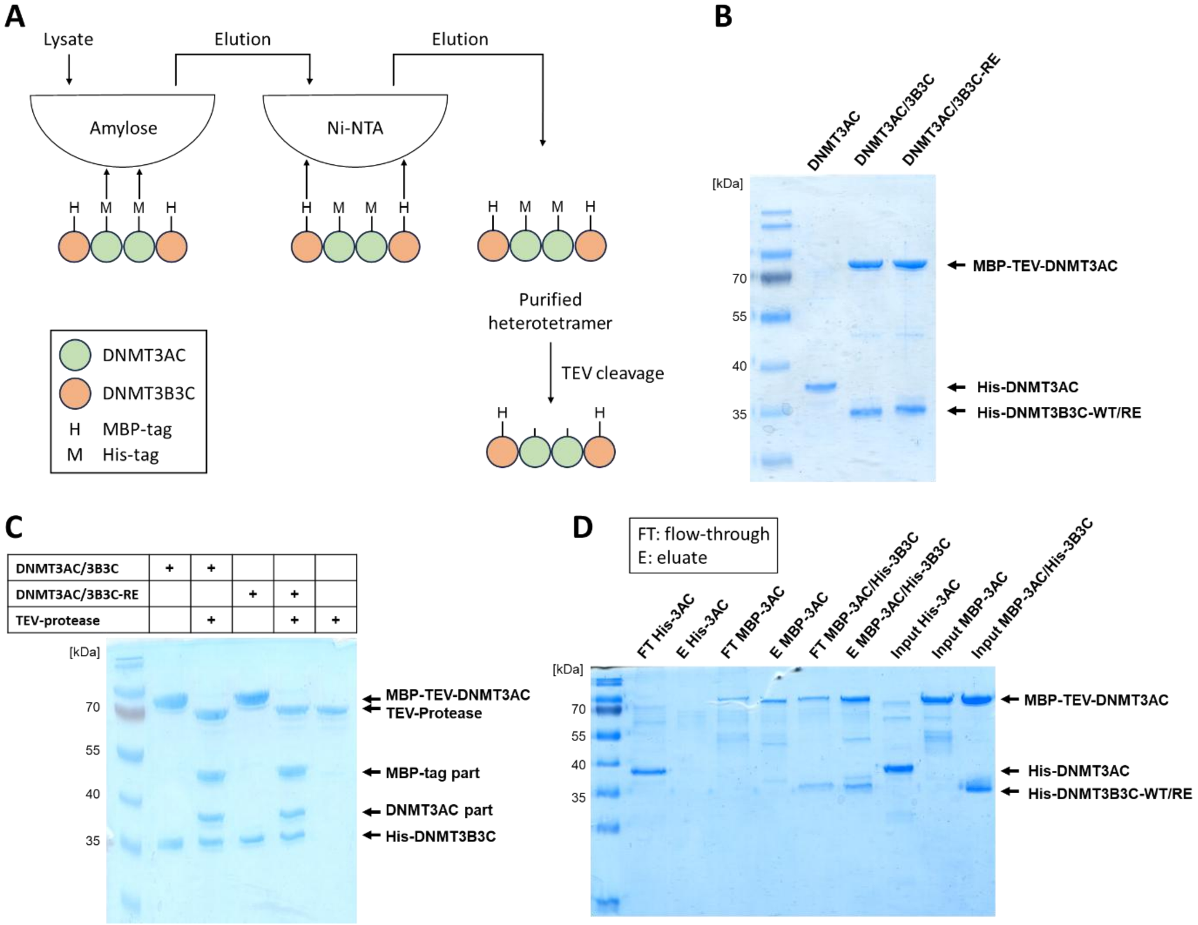

Figure S3: Quality control and processing steps of the nucleosome methylation experiments. A) Distribution of errors of linker methylation levels determined in the Nuc1-linker and linker-Nuc2 sequencing analyses in all experiments. **B)** Methylation levels observed in no-enzyme controls in all experiments. In both panels, values for individual CpG sites are shown.

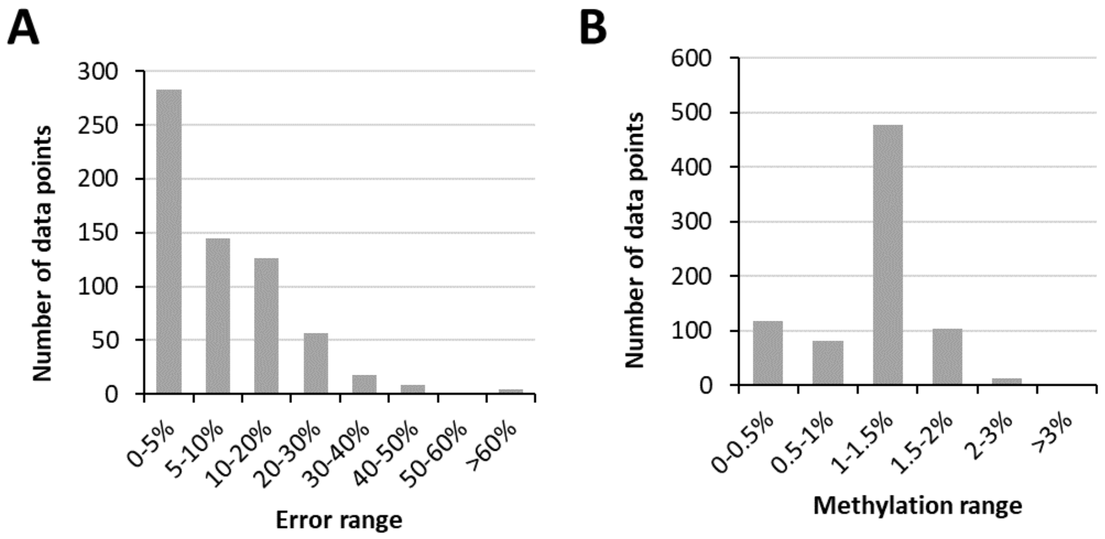

Figure S4: Exemplary data set showing the methylation levels determined in the competitive methylation of all three dinucleosome substrates. A, B) Methylation in the top and bottom DNA strands of the dinucleosomal Linker-70 substrate. **C, D)** Methylation in the top and bottom DNA strands of the free DNA substrate.

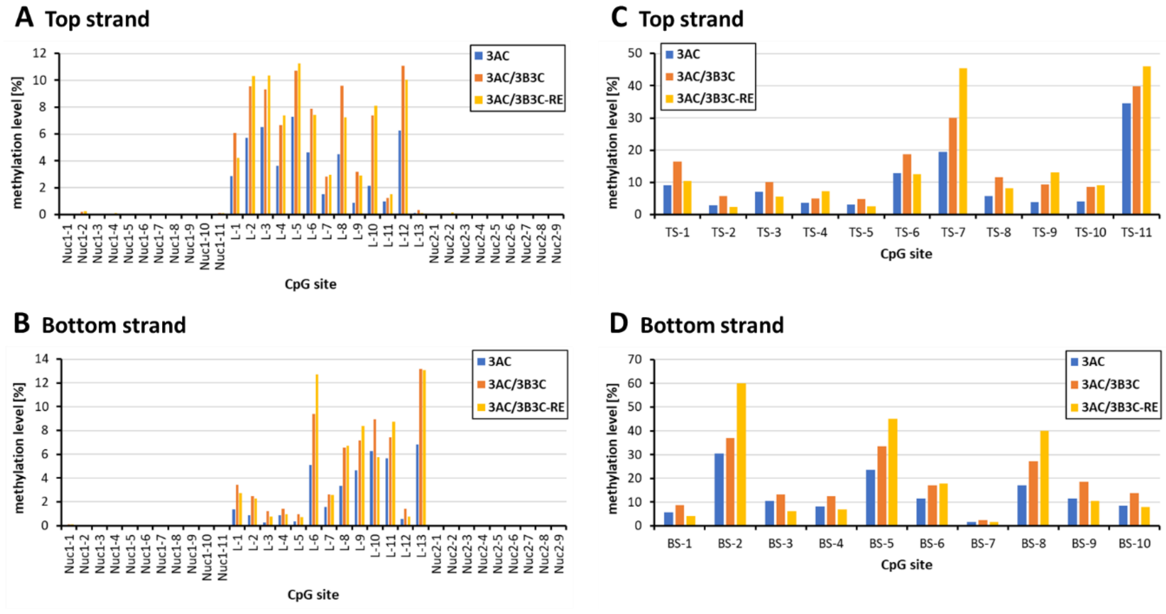

Figure S5: Additional information related to the competitive methylation of nucleosomal and free DNA by DNMT3AC/3B3C. A) Sequence and CpG site annotation of the free DNA used in the competitive methylation experiments. **B)** Scatter plot showing the correlation of DNMT3AC/3B3C activity in methylation of free DNA and DNMT3AC flanking sequence preferences displayed in Figure 4C. **C)** Same as panel B but showing the correlation of DNMT3AC/3B3C activity in methylation of nucleosomal linker DNA and DNMT3AC flanking sequence preferences displayed in Figure 4C.

A

1 2 3 4 5 6 7

CTATGGAAACCCCTGTGGAGCTTCAGGGGCACGAGTGAGG CGGGCGCTGGCGGGCCAAGGTGACGAAGGCGCCTCCGGC

8 9 10 11

TCTTGGGCCAGCGGACTGAGCGGTGGAGCAGAACTTGGGTGCCTCGGGGACCGCCAAAAAGTGGCCTTGTCCACTTCTCTGAG

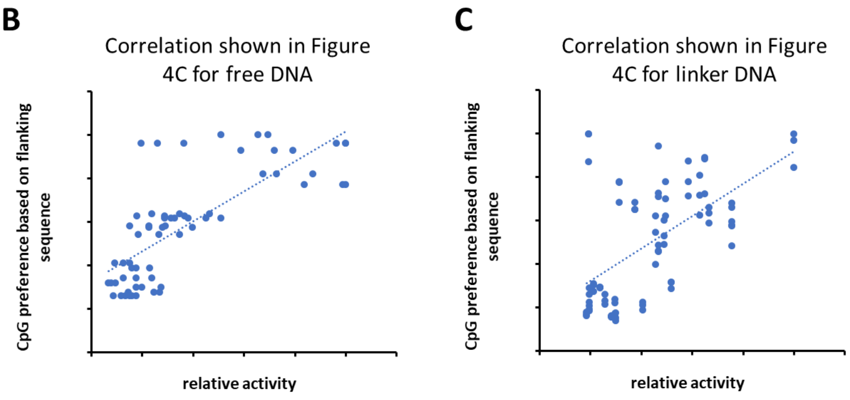

Figure S6: Exemplary data set showing the methylation levels in the top and bottom DNA strands determined in the competitive methylation of dinucleosome substrates with free DNA.

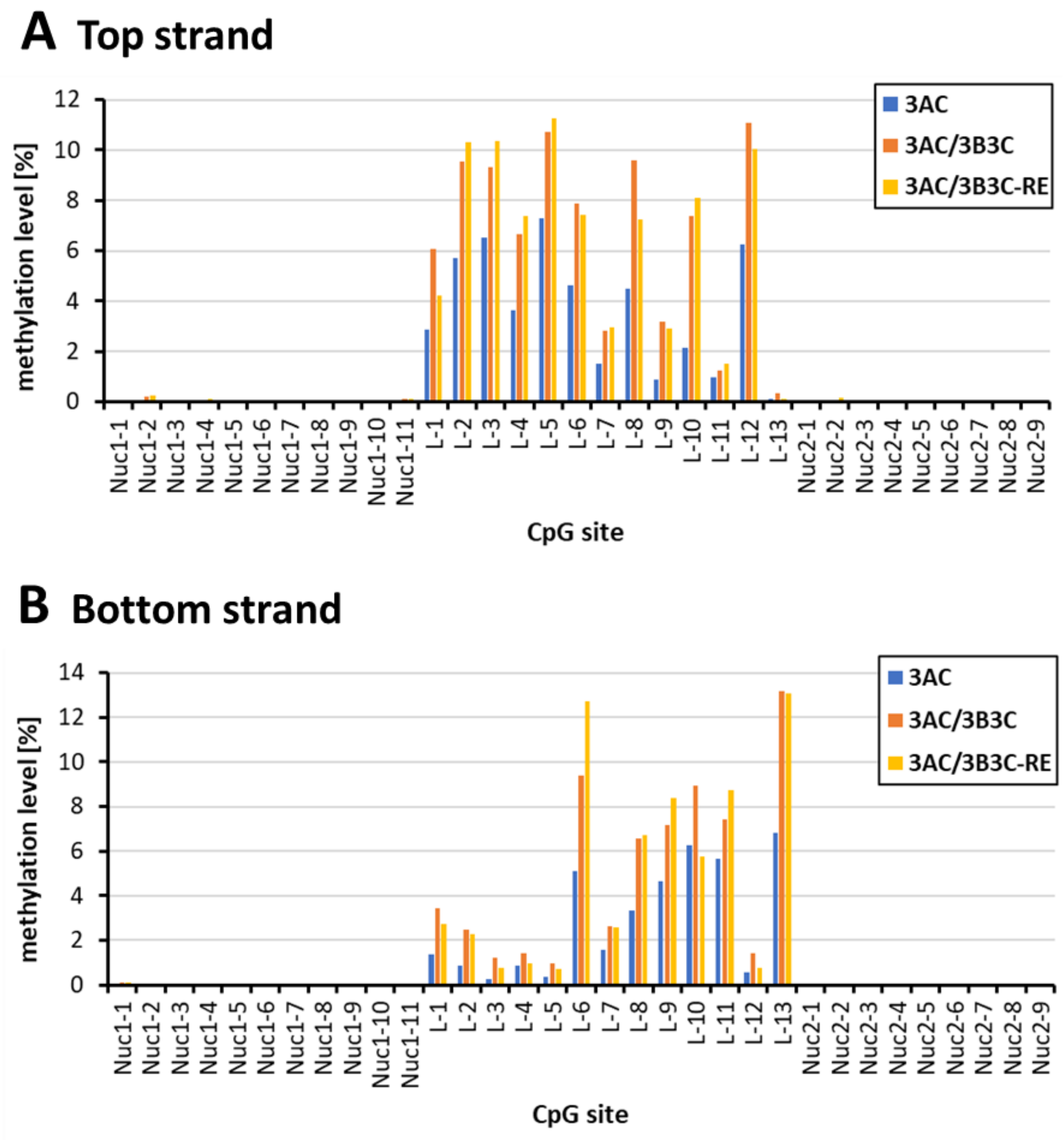

Figure S7: Individual data points of the relative DNMT3AC/3B3C activity at all CpG sites in the Linker-70 dinucleosome. The averages and standard deviations shown in Figure 5A are indicated.

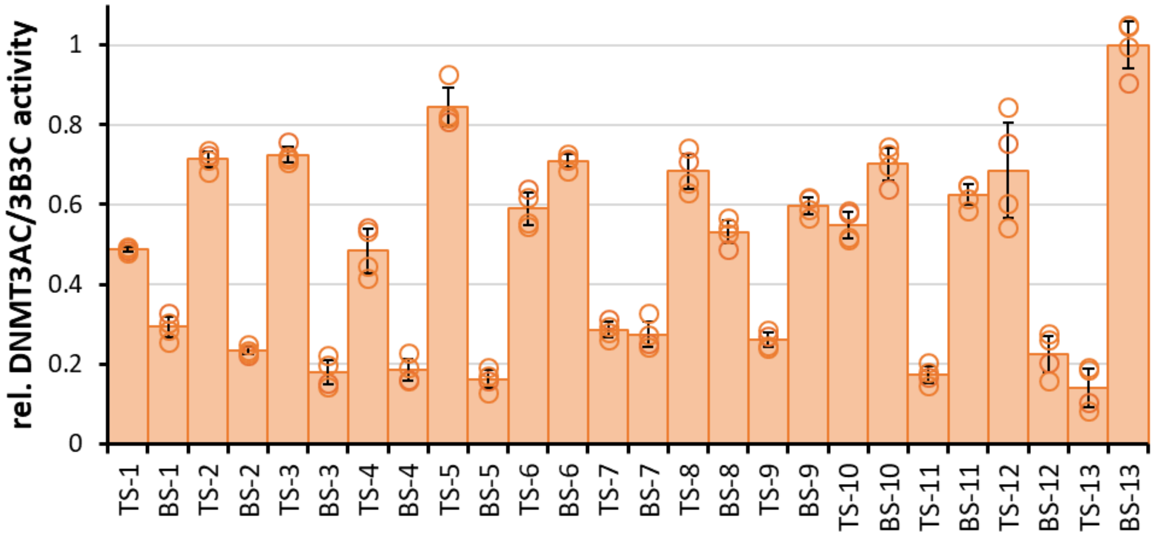

Text S1: DNA sequence of the dinucleosome substrate with Linker-70.

> Dinucleosome Linker-70

CGAGGTCGACGGTATCGATAAGCTTCTGGAGAATCCCCCAGCCGAGGCCGCTCAATTGGTCGTAGCAAGCTCT
AGCACCGCTTAAACGCACGTACGCGTTGTCCCCCGCGTTTTAACCGCCAAGGGGATTACTCCCTAGTCTCCAGG
CAGGTGTCAGATATATACATCCTGTAACGCTATCCGCGCCACGTCTACGCTNNNTACGAGAACGCCGAGACGT
GCGAGCAGCGAAAGCGGCCGaCCTGGAGAATCCAGGTGCTGAGGCAGCTCAATTGGTCGTAGCAAGCTCTAG
CACCGCTTAAACGCACGTACGCGTTGTCCCCCGCGTTTTAACCGCCAAGGGGATTACTCCCTAGTCTCCAGGCA
CCACTCAGATATATACATCCTGTAAGGGCGAATTCCACATTG

Text S2: DNA sequence of the dinucleosome substrate with Linker-58(1).

> Dinucleosome Linker-58(1)

CGAGGTCGACGGTATCGATAAGCTTCTGGAGAATCCCCCAGCCGAGGCCGCTCAATTGGTCGTAGCAAGCTCT
AGCACCGCTTAAACGCACGTACGCGTTGTCCCCCGCGTTTTAACCGCCAAGGGGATTACTCCCTAGTCTCCAGG
CAGGTGTCAGATATATACATCCTGTGCCACGTCTACGCTNNNTACGAGAACGCCGAGACGTGCGAGCAGCGA
AAGCGGCCGaCCTGGAGAATCCAGGTGCTGAGGCAGCTCAATTGGTCGTAGCAAGCTCTAGCACCGCTTAAAC
GCACGTACGCGTTGTCCCCCGCGTTTTAACCGCCAAGGGGATTACTCCCTAGTCTCCAGGCACCACTCAGATAT
ATACATCCTGTAAGGGCGAATTCCACATTG

Text S3: DNA sequence of the dinucleosome substrate with Linker-58(2).

> Dinucleosome Linker-58(2)

CGAGGTCGACGGTATCGATAAGCTTCTGGAGAATCCCCCAGCCGAGGCCGCTCAATTGGTCGTAGCAAGCTCT
AGCACCGCTTAAACGCACGTACGCGTTGTCCCCCGCGTTTTAACCGCCAAGGGGATTACTCCCTAGTCTCCAGG
CAGGTGTCAGATATATACATCCTGTAACGCTATCCGCGCCACGTCTACGCTNNNGAGACGTGCGAGCAGCGAA
AGCGGCCGaCCTGGAGAATCCAGGTGCTGAGGCAGCTCAATTGGTCGTAGCAAGCTCTAGCACCGCTTAAACG
CACGTACGCGTTGTCCCCCGCGTTTTAACCGCCAAGGGGATTACTCCCTAGTCTCCAGGCACCACTCAGATATA
TACATCCTGTAAGGGCGAATTCCACATTG

Text S4: DNA sequence of the CpG-rich DNA.

> CpG-rich DNA

CTATGGAAACCCCTGTGGAGCTTCAGGGGCACGAGTGAGGCGGGCGCTGGCGGGCCAAGGTGACGAAGGCG
CCTCCGGCTCTTGGGCCAGCGGACTGAGCGGTGGAGCAGAACTTGGGTGCCTCGGGGACCGCCAAAAAGTGG
CCTTGTCCACTTCTCTGAG

Text S5: Protein sequence of the His-DNMT3AC used in methylation assays.

> His-DNMT3AC

MGSSHHHHHHSSGLVPRGSHMNHDQEFDPPKVYPPVPAEKRKPIRVLSLFDGIATGLLVLKDLGIQVDRYIASEVCE
DSITVGMVRHQGKIMYVGDVRSVTQKHIQEWGPFDLVIGGSPCNDLSIVNPARKGLYEGTGRLFFEFYRLLHDARP
KEGDDRPFFWLFENVVAMGVSDKRDISRFLESNPVMIDAKEVSAAHRARYFWGNLPGMNRPLASTVNDKLELQE
CLEHGRIAKFSKVRTITTRSNSIKQGKDQHFPVFMNEKEDILWCTEMERVFGFPVHYTDVSNMSRLARQRLLGRSW
SVPVIRHLFAPLKEYFACV*

Text S6: Protein sequence of the MBP-TEV-DNMT3AC used in methylation assays.

> MBP-TEV-DNMT3AC

MKIEEGKLVIWINGDKGYNGLAEVGKKFEKDTGIKVTVEHPDKLEEKFPQVAATGDGPDIIFWAHDRFGGYAQSGL
LAEITPDKAFQDKLYPFTWDAVRYNGKLIAYPIAVEALSLIYNKDLLPNPPKTWEEIPALDKELKAKGKSALMFNLQEP
YFTWPLIAADGGYAFKYENGKYDIKDVGVDNAGAKAGLTFLVDLIKNKHMNADTDYSIAEAAFNKGETAMTINGP
WAWSNIDTSKVNYGVTVLPTFKGQPSKPFVGVLSAGINAASPNKELAKEFLENYLLTDEGLEAVNKDKPLGAVALKS
YEEELAKDPRIAATMENAQKGEIMPNIPQMSAFWYAVRTAVINAASGRQTVDEALKDAQTNSSSNNNNNNNNN
NLGIEGRENLYFQGHMNHDQEFDPPKVYPPVPAEKRKPIRVLSLFDGIATGLLVLKDLGIQVDRYIASEVCEDSITVG
MVRHQGKIMYVGDVRSVTQKHIQEWGPFDLVIGGSPCNDLSIVNPARKGLYEGTGRLFFEFYRLLHDARPKEGDD
RPFFWLFENVVAMGVSDKRDISRFLESNPVMIDAKEVSAAHRARYFWGNLPGMNRPLASTVNDKLELQECLEHG
RIAKFSKVRTITTRSNSIKQGKDQHFPVFMNEKEDILWCTEMERVFGFPVHYTDVSNMSRLARQRLLGRSWSVPVI
RHLFAPLKEYFACV*

Text S7: Protein sequence of the His-DNMT3B3C used in methylation assays.

> His-DNMT3B3C

MGSSHHHHHHSSGLVPRGSHMDPDLEEFEPPKLYPAIPAAKRRPIRVLSLFDGIATGYLVLKELGIKVEKYIASEVCAE
SIAVGTVKHEGQIKYVNDVRKITKKNIEEWGPFDLVIGGSPCNDLSNVNPARKGLYEGTGRLFFEFYHLLNYTRPKEG
DNRPFFWMFENVVAMKVNDKKDISRFLACNPVMIDAIKVSAAHRARYFWGNLPGMNRIFGFPAHYTDVSNMGR
GARQKLLGRSWSVPVIRHLFAPLKDYFACE*

Text S8: Protein sequence of the His-DNMT3B3C-RE used in methylation assays.

> His-DNMT3B3C-RE

MGSSHHHHHHSSGLVPRGSHMDPDLEEFEPPKLYPAIPAAKRRPIRVLSLFDGIATGYLVLKELGIKVEKYIASEVCAE
SIAVGTVKHEGQIKYVNDVRKITKKNIEEWGPFDLVIGGSPCNDLSNVNPARKGLYEGTGRLFFEFYHLLNYTRPKEG
DNRPFFWMFENVVAMKVNDKKDISRFLACNPVMIDAIKVSAAHRARYFWGNLPGMNRIFGFPAHYTDVSNMGE
GAEQKLLGRSWSVPVIRHLFAPLKDYFACE*

Table S1: List of primers used for DNMT3B3 mutagenesis

Name	Sequence [5'-3']
DNMT3B3C_RE_FP	AACATGGGCGAAGGCGCCGAACAGAAGCTGCT
DNMT3B3C_RE_RP	GGACACGTCCGTGTAGT

Table S2: List of primers used for the generation of the dinucleosome DNAs

Name	Sequence [5'-3']
601_MluI_FP	CGCACGTACGCGTTGTCCCCCGCGT
601_Nuc2_FP	GAGAATCCAGGTGCTGAGGCAGCTCAATTGG
601_Nuc2_RP	GGATGTATATATCTGAGTGGTGCCTGGAGACTAGGG
601_Nuc1_FP	TGGAGAATCCCCCAGCCGAGGCCGC
601_Nuc1_RP	GTATATATCTGACACCTGCCTGGAGACTAGGG
601_Nuc2_70bpLinker_FP	AACGCTATCCGCGCCACGTCTACGCTNNNTACGAGAACGCCGAGACGTGCG
601_Nuc2_58(1)Linker_FP	GCCACGTCTACGCTNNNTACGAGAACGCCGAGACGTGCG
601_Nuc2_58(2)Linker_FP	AACGCTATCCGCGCCACGTCTACGCTNNNGAGACGTGCGAGCAG
601_Nuc2_Linker_RP	ACAGGATGTATATATCTGAGTGGTGCC
Vector_601_Nuc2_70bpLinker_FP	TATATACATCCTGTAACGCTATCCGCGCCACGTCTAC
Vector_601_Nuc2_58(1)Linker_FP	TATATACATCCTGTGCCACGTCTACGCT
Vector_601_Nuc2_58(2)Linker_FP	TATATACATCCTGTAACGCTATCCGCGCCAC
Vector_601_Nuc2_RP	GGGGGATTCTCCAGAAGCTTATCGATACCGTCGACC
Fragment_Nuc1_70bpLinker_FP	TATCGATAAGCTTCTGGAGAATCCCCCAGCCG
Fragment_Nuc1_70bpLinker_RP	GCGCGGATAGCGTTACAGGATGTATATATCTGACACCTGCCTGGAG
Fragment_Nuc1_58(1)Linker_RP	AGCGTAGACGTGGCACAGGATGTATATATCTGACACCTGCCTGGAG
Fragment_Nuc1_58(2)Linker_RP	GTGGCGCGGATAGCGTTACAGGATGTATATATCTGACACCTGCCTGGAG

Table S3: List of primers used for sequencing

Barcode NGS

BC	Forward Primer	Reverse Primer
1	ACACTCTTTCCCTACACGACGCTCTTCCGATCTNNNNNNATAGATAAGTTTTTGGAGAATTTTTTAGT	GTGACTGGAGTTCAGACGTGTGCTCTTCCGATCTNNNNNATAACCAATTAAACTACCTCAACACC
2	ACACTCTTTCCCTACACGACGCTCTTCCGATCTNNNNNNTACGATAAGTTTTTGGAGAATTTTTTAGT	GTGACTGGAGTTCAGACGTGTGCTCTTCCGATCTNNNNNTACACCAATTAAACTACCTCAACACC
3	ACACTCTTTCCCTACACGACGCTCTTCCGATCTNNNNNNGTGGATAAGTTTTTGGAGAATTTTTTAGT	GTGACTGGAGTTCAGACGTGTGCTCTTCCGATCTNNNNNGTGACCAATTAAACTACCTCAACACC
4	ACACTCTTTCCCTACACGACGCTCTTCCGATCTNNNNNNCTCGATAAGTTTTTGGAGAATTTTTTAGT	GTGACTGGAGTTCAGACGTGTGCTCTTCCGATCTNNNNNCTCACCAATTAAACTACCTCAACACC
5	ACACTCTTTCCCTACACGACGCTCTTCCGATCTNNNNNNACGGATAAGTTTTTGGAGAATTTTTTAGT	GTGACTGGAGTTCAGACGTGTGCTCTTCCGATCTNNNNNACGACCAATTAAACTACCTCAACACC
6	ACACTCTTTCCCTACACGACGCTCTTCCGATCTNNNNNNTGTGATAAGTTTTTGGAGAATTTTTTAGT	GTGACTGGAGTTCAGACGTGTGCTCTTCCGATCTNNNNNTGTACCAATTAAACTACCTCAACACC
7	ACACTCTTTCCCTACACGACGCTCTTCCGATCTNNNNNNATAGGGGATTATTTTTTAGTTTTTAGGTAGGTG	GTGACTGGAGTTCAGACGTGTGCTCTTCCGATCTNNNNNATAACCCTTACAAAATATATATATCTAAATAATACC
8	ACACTCTTTCCCTACACGACGCTCTTCCGATCTNNNNNNTACGGGGATTATTTTTTAGTTTTTAGGTAGGTG	GTGACTGGAGTTCAGACGTGTGCTCTTCCGATCTNNNNNTACACCCTTACAAAATATATATATCTAAATAATACC
9	ACACTCTTTCCCTACACGACGCTCTTCCGATCTNNNNNNGTGGGGGATTATTTTTTAGTTTTTAGGTAGGTG	GTGACTGGAGTTCAGACGTGTGCTCTTCCGATCTNNNNNGTGACCCTTACAAAATATATATATCTAAATAATACC
10	ACACTCTTTCCCTACACGACGCTCTTCCGATCTNNNNNNCTCGGGGATTATTTTTTAGTTTTTAGGTAGGTG	GTGACTGGAGTTCAGACGTGTGCTCTTCCGATCTNNNNNCTCACCCTTACAAAATATATATATCTAAATAATACC
11	ACACTCTTTCCCTACACGACGCTCTTCCGATCTNNNNNNACGGGGGATTATTTTTTAGTTTTTAGGTAGGTG	GTGACTGGAGTTCAGACGTGTGCTCTTCCGATCTNNNNNACGACCCTTACAAAATATATATATCTAAATAATACC
12	ACACTCTTTCCCTACACGACGCTCTTCCGATCTNNNNNNTGTGGGGATTATTTTTTAGTTTTTAGGTAGGTG	GTGACTGGAGTTCAGACGTGTGCTCTTCCGATCTNNNNNTGTACCCTTACAAAATATATATATCTAAATAATACC
13	ACACTCTTTCCCTACACGACGCTCTTCCGATCTNNNNNATATCGATTAATTGAGTTGTTTTAGTATT	GTGACTGGAGTTCAGACGTGTGCTCTTCCGATCTNNNNNATAAATAAACTTCTAAAAAATCCCCCAAC
14	ACACTCTTTCCCTACACGACGCTCTTCCGATCTNNNNNTATACGATTAATTGAGTTGTTTTAGTATT	GTGACTGGAGTTCAGACGTGTGCTCTTCCGATCTNNNNNTACAATAAACTTCTAAAAAATCCCCCAAC
15	ACACTCTTTCCCTACACGACGCTCTTCCGATCTNNNNNCAGTAGATTAATTGAGTTGTTTTAGTATT	GTGACTGGAGTTCAGACGTGTGCTCTTCCGATCTNNNNNGTGAATAAACTTCTAAAAAATCCCCCAAC
16	ACACTCTTTCCCTACACGACGCTCTTCCGATCTNNNNNATAGTTTTTATAGGATGTATATATTTGAGTGGTGTT	GTGACTGGAGTTCAGACGTGTGCTCTTCCGATCTNNNNNAGCGACAAAAAATTACTCCCTAATCTCCAAACAAATA
17	ACACTCTTTCCCTACACGACGCTCTTCCGATCTNNNNNTACGTTTTTATAGGATGTATATATTTGAGTGGTGTT	GTGACTGGAGTTCAGACGTGTGCTCTTCCGATCTNNNNNTCGACCAAAAAATTACTCCCTAATCTCCAAACAAATA

18	ACACTCTTTCCCTACACGACGCTCTT CCGATCTNNNNNGTGGTTTTTATAGG ATGTATATATTTGAGTGGTGTT	GTGACTGGAGTTCAGACGTGTGCTCT TCCGATCTNNNNNCTGTCTAAAAAATT ACTCCCTAATCTCCAAACAAATA
19	ACACTCTTTCCCTACACGACGCTCTT CCGATCTNNNNNNATATTATGGAAAT TTTTGTGGAGTTTTAGGG	GTGACTGGAGTTCAGACGTGTGCTCT TCCGATCTNNNNNATACTCAAAAAAAT AAACAAAACCACTTTTTAAC
20	ACACTCTTTCCCTACACGACGCTCTT CCGATCTNNNNNNTACTTATGGAAAT TTTTGTGGAGTTTTAGGG	GTGACTGGAGTTCAGACGTGTGCTCT TCCGATCTNNNNNTACCTCAAAAAAAT AAACAAAACCACTTTTTAAC
21	ACACTCTTTCCCTACACGACGCTCTT CCGATCTNNNNNNGTGTTATGGAAAT TTTTGTGGAGTTTTAGGG	GTGACTGGAGTTCAGACGTGTGCTCT TCCGATCTNNNNNGTGCTCAAAAAAA TAAACAAAACCACTTTTTAAC
19	ACACTCTTTCCCTACACGACGCTCTT CCGATCTNNNNNATATTTAGAGAAGT GGATAAGGTTATTTTTTGGT	GTGACTGGAGTTCAGACGTGTGCTCT TCCGATCTNNNNNATAAAAAACCCCT ATAAAACTTCAAAAACAC
20	ACACTCTTTCCCTACACGACGCTCTT CCGATCTNNNNNTACTTTAGAGAAGT GGATAAGGTTATTTTTTGGT	GTGACTGGAGTTCAGACGTGTGCTCT TCCGATCTNNNNNTACAAAAACCCCT ATAAAACTTCAAAAACAC
21	ACACTCTTTCCCTACACGACGCTCTT CCGATCTNNNNNGTGTTTAGAGAAGT GGATAAGGTTATTTTTTGGT	GTGACTGGAGTTCAGACGTGTGCTCT TCCGATCTNNNNNGTGAAAAACCCCT ATAAAACTTCAAAAACAC

Index NGS

I	Forward Primer	Reverse Primer
1	AATGATACGGCGACCACCGAGATCT ACACATTACTCGACACTCTTTCCCTA CACGACGCTCTTCCGATCT	CAAGCAGAAGACGGCATACGAGAT CGAGTAATGTGACTGGAGTTCAGAC GTGTGCTCTTCCGATCT
2	AATGATACGGCGACCACCGAGATCT ACACTCCGGAGAACACTCTTTCCCTA CACGACGCTCTTCCGATCT	CAAGCAGAAGACGGCATACGAGAT TCTCCGGAGTGACTGGAGTTCAGA CGTGTGCTCTTCCGATCT
3	AATGATACGGCGACCACCGAGATCT ACACCGCTCATTACACTCTTTCCCTA CACGACGCTCTTCCGATCT	CAAGCAGAAGACGGCATACGAGAT AATGAGCGGTGACTGGAGTTCAGA CGTGTGCTCTTCCGATCT
4	AATGATACGGCGACCACCGAGATCT ACACGAGATTCCACACTCTTTCCCTA CACGACGCTCTTCCGATCT	CAAGCAGAAGACGGCATACGAGAT GGAATCTCGTGACTGGAGTTCAGAC GTGTGCTCTTCCGATCT
5	AATGATACGGCGACCACCGAGATCT ACACATTCAGAAACACTCTTTCCCTA CACGACGCTCTTCCGATCT	CAAGCAGAAGACGGCATACGAGAT TTCTGAATGTGACTGGAGTTCAGAC GTGTGCTCTTCCGATCT
6	AATGATACGGCGACCACCGAGATCT ACACGAATTCGTACACTCTTTCCCTA CACGACGCTCTTCCGATCT	CAAGCAGAAGACGGCATACGAGAT ACGAATTCGTGACTGGAGTTCAGAC GTGTGCTCTTCCGATCT
7	AATGATACGGCGACCACCGAGATCT ACACCTGAAGCTACACTCTTTCCCTA CACGACGCTCTTCCGATCT	CAAGCAGAAGACGGCATACGAGAT AGCTTCAGGTGACTGGAGTTCAGAC GTGTGCTCTTCCGATCT
8	AATGATACGGCGACCACCGAGATCT ACACTAATGCGCACACTCTTTCCCTA CACGACGCTCTTCCGATCT	CAAGCAGAAGACGGCATACGAGAT GCGCATTAGTGACTGGAGTTCAGAC GTGTGCTCTTCCGATCT
9	AATGATACGGCGACCACCGAGATCT ACACCGGCTATGACACTCTTTCCCTA CACGACGCTCTTCCGATCT	CAAGCAGAAGACGGCATACGAGAT CATAGCCGGTGACTGGAGTTCAGA CGTGTGCTCTTCCGATCT
10	AATGATACGGCGACCACCGAGATCT ACACTCCGCGAAACACTCTTTCCCTA CACGACGCTCTTCCGATCT	CAAGCAGAAGACGGCATACGAGAT TTCGCGGAGTGACTGGAGTTCAGA CGTGTGCTCTTCCGATCT

11	AATGATACGGCGACCACCGAGATCT ACACTCTCGCGCACACTCTTTCCCTA CACGACGCTCTTCCGATCT	CAAGCAGAAGACGGCATACGAGAT GCGCGAGAGTGACTGGAGTTCAGA CGTGTGCTCTTCCGATCT
12	AATGATACGGCGACCACCGAGATCT ACACAGCGATAGACACTCTTTCCCTA CACGACGCTCTTCCGATCT	CAAGCAGAAGACGGCATACGAGAT CTATCGCTGTGACTGGAGTTCAGAC GTGTGCTCTTCCGATCT
13	AATGATACGGCGACCACCGAGATCT ACACTTCCTCCTACACTCTTTCCCTA CACGACGCTCTTCCGATCT	CAAGCAGAAGACGGCATACGAGAT AGGAGGAAGTGACTGGAGTTCAGA CGTGTGCTCTTCCGATCT
14	AATGATACGGCGACCACCGAGATCT ACACTGCTTGCTACACTCTTTCCCTA CACGACGCTCTTCCGATCT	CAAGCAGAAGACGGCATACGAGAT AGCAAGCAGTGACTGGAGTTCAGA CGTGTGCTCTTCCGATCT
